# FG repeats drive co-clustering of nuclear pores and P granules in the *C. elegans* germline

**DOI:** 10.1242/dev.204585

**Published:** 2025-03-27

**Authors:** Laura L. Thomas, Devavrat M. Bodas, Geraldine Seydoux

**Affiliations:** HHMI and Department of Molecular Biology and Genetics, Johns Hopkins University School of Medicine, Baltimore, MD 21205, USA

**Keywords:** Nuclear pore, P granule, Germ granule, Histone, *C*. *elegans*

## Abstract

Condensates that accumulate small RNA biogenesis factors (nuage) are common in germ cells and often associate with nuclei. In the *Caenorhabditis elegans* germline, P granules overlay large clusters of nuclear pores and this organization has been proposed to facilitate surveillance of nascent transcripts by Argonaute proteins enriched in P granules. We report that co-clustering of nuclear pores and P granules depends on FG repeat-containing nucleoporins and FG repeats in the Vasa class helicase GLH-1. Worms with mutations that prevent this co-clustering are fertile under standard growth conditions and exhibit misregulation of only a minority of genes, including replication-dependent histones. Our observations suggest that association with nuclear pores, although non-essential for genome surveillance, may serve to tune mRNA flow through P granules and other nuage condensates.

## Teaser

**Summary:** P granule co-clustering with nuclear pores in the *Caenorhabditis elegans* germline is mediated by FG repeats and is not essential for genome surveillance.

## INTRODUCTION

A hallmark of germ cells is the presence of germ granules, biomolecular condensates containing protein and RNA. Germ granules often associate with the nuclear envelope and perinuclear granules have been reported in a wide range of species including *Drosophila*, *Xenopus*, zebrafish and mice (Chuma et al., 2009; Aravin et al., 2009; Knaut et al., 2000; Eddy, 1975; Eddy and Ito, 1971; Hay et al., 1988). In the model system *Caenorhabditis elegans*, germ granules called P granules stably associate with the nuclear envelope in germ cell progenitors and germ cells in early meiotic prophase (pachytene) (Strome and Wood, 1982; Hird et al., 1996; Updike and Strome, 2010). Seminal studies reported that, in the pachytene germline, P granules overlay large clusters of nuclear pores (Pitt et al., 2000; Sheth et al., 2010). Subsequent studies identified several nuclear pore proteins required for wild-type P granule morphology and/or association with the nuclear envelope (Updike and Strome, 2009; Voronina and Seydoux, 2010). Here, we address the molecular requirements for the co-clustering of P granules with nuclear pores.

Nuclear pores are highly conserved structures that contain at least 30 different proteins called nucleoporins (Nups) (Hampoelz et al., 2019; Cohen-Fix and Askjaer, 2017). Approximately two-thirds of Nups are core structural components that scaffold the pore. The remaining one-third localize to the central channel, the nuclear basket and/or cytoplasmic filaments and contain large, intrinsically disordered domains rich in phenylalanine and glycine (FG domains). FG-Nups have been shown to interact in a cohesive manner both *in vitro* and *in vivo* (Xu and Powers, 2013; Labokha et al., 2012; Frey et al., 2006; Hülsmann et al., 2012; Schmidt and Görlich, 2015; Patel et al., 2007; Ader et al., 2010). Cohesive interactions among FG-Nups have been proposed to form a phase-separated network that constitutes the permeability barrier that separates the nucleoplasm from the cytoplasm (Ribbeck and Görlich, 2001; Schmidt and Görlich, 2016). Several P granule proteins also contain FG repeats, and perinuclear P granules have been proposed to function as cytoplasmic extensions of nuclear pores (Sheth et al., 2010; Updike et al., 2011).

Electron microscopy studies estimated that ∼75% of nuclear pores are covered by P granules in the *C. elegans* pachytene germline (Pitt et al., 2000). Experiments using heat-induced transcripts revealed that nascent mRNAs accumulate briefly in perinuclear granules before entering the cytoplasm (Sheth et al., 2010). P granule proteins are essential for the maintenance of germ cell identity and fertility: co-depletion of key P granule proteins causes misexpression of somatic genes in germ cells and highly penetrant sterility (Updike et al., 2014; Knutson et al., 2017; Ciosk et al., 2006). P granules contain multiple Argonaute proteins complexed with small RNAs (sRNAs) antisense to mRNAs. An often-cited model is that clustering of nuclear pores under P granules allows for nascent transcripts to be scanned by Argonaute/sRNA complexes and silenced or licensed for expression before release to the cytoplasm (Sundby et al., 2021; Phillips and Updike, 2022).

We have systematically investigated the requirements and consequences of the association between nuclear pores and P granules in the *C. elegans* germline. We find that clustering of nuclear pores depends on FG-Nups and FG repeats in the P granule protein GLH-1. We generate mutants that uncouple the connection between nuclear pores and P granules and find, surprisingly, that these mutants are fertile under standard laboratory growth conditions and misregulate only a subset of transcripts. Our observations suggest that co-clustering of nuclear pores and P granules tunes RNA dynamics in P granules but may not be required for the activity of most P granule proteins and the regulation of most genes under standard growth conditions.

## RESULTS

### Nuclear pores form clusters in immature germ cells

**Fig. 1.**
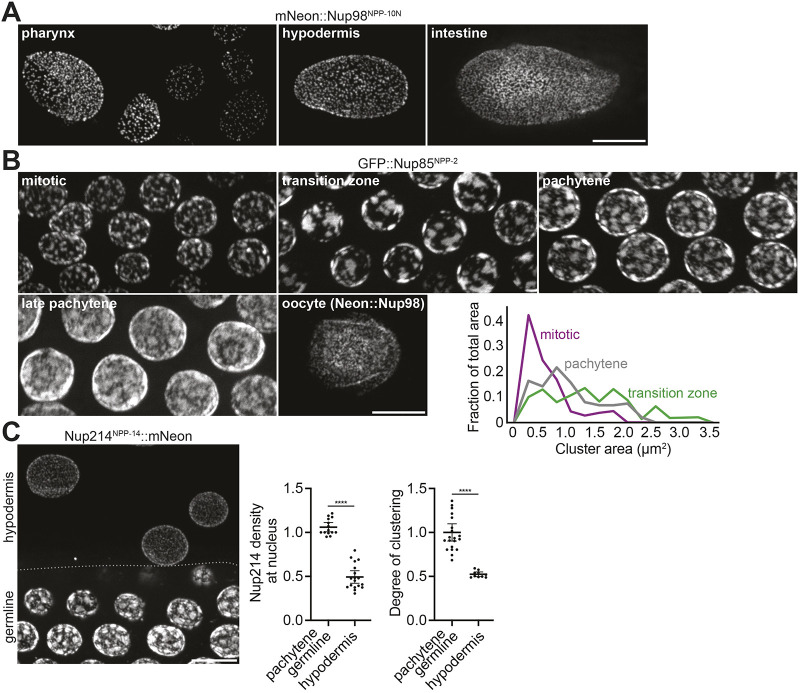
**Nuclear pores form clusters in the *C. elegans* germline.** (A) Representative confocal micrographs of nuclei expressing mNeonGreen::Nup98^NPP-10N^ in *C. elegans* pharynx, hypodermal and intestinal cells. (B) Left: Representative confocal micrographs of nuclei expressing GFP::Nup85^NPP-2^ or mNeonGreen::Nup98^NPP-10N^ in different regions of the germline. Bottom right: Frequency graph plotting the size distribution of nuclear pore clusters for each germline region, binned by cluster area (generated from *n*≥323 clusters). (C) Left: Representative confocal micrograph of nuclei expressing Nup214^NPP-14^::mNeonGreen in hypodermal cells and the pachytene germline. Middle: Normalized Nup214^NPP-14^::mNeonGreen signal per area in germline and hypodermal nuclei. Error bars represent 95% CI for *n*≥14 nuclei. Right: Normalized pore clustering in germline versus hypodermal nuclei. Error bars represent 95% CI for *n*≥10 nuclei. Each measurement represents a single nucleus. All images are maximum intensity projections. ****P<0.0001 (unpaired *t*-test). Scale bars: 5 µm.

To quantify systematically the distribution of nuclear pores in nuclei across different tissues, we imaged mNeonGreen::Nup98 (*C. elegans* NPP-10N), GFP::Nup85 (NPP-2), Nup214 (NPP-14)::mNeonGreen and GFP::NDC1 (NPP-22) fusions (tagged at the endogenous loci) in adult hermaphrodites and embryos. Nuclei in somatic tissues showed a uniform distribution of nuclear pores across the nuclear envelope, similar to what has been observed in tissue culture cells (Fig. 1A) (Daigle et al., 2001; Rabut et al., 2004; Dultz and Ellenberg, 2010; Thevathasan et al., 2019; Cheng et al., 2021; Xie et al., 2016). In contrast, as reported previously (Pitt et al., 2000; Sheth et al., 2010), we observed prominent clustering of nuclear pores in the germline (Fig. 1B,C). The germline of adult hermaphrodites is organized along two tube-shaped syncytia in which nuclei progress from mitosis at the distal end to prophase of meiosis I before becoming enclosed by the plasma membrane to form individual oocytes at the proximal end (Fig. S1A) (Hubbard, 2005; Kimble, 2005). We observed small clusters of nuclear pores (∼0.5 µm^2^) in the mitotic zone at the distal end that grew larger upon entry into meiosis (>2 µm^2^) and were maintained throughout the pachytene region (Fig. 1B). Pore clustering diminished in late pachytene and disappeared in oocytes (Fig. 1B). Pore clustering was absent from the earliest germline precursors in embryos and reappeared in the germline founder cell P_4_ and its two daughters the primordial germ cells Z2 and Z3 (Fig. S1B).

We confirmed nuclear pore clustering in pachytene nuclei using eight additional nuclear pore markers tagged at the endogenous loci (Fig. S1C). Nuclear pore density was higher in germline nuclei compared to somatic nuclei (Fig. 1C), raising the possibility that clustering may be a consequence of high pore density. We conclude that pore clustering is a property of immature germ cells with elevated pore density and is most prominent during pachytene.

### Nuclear pore clusters colocalize with P granules

**Fig. 2.**
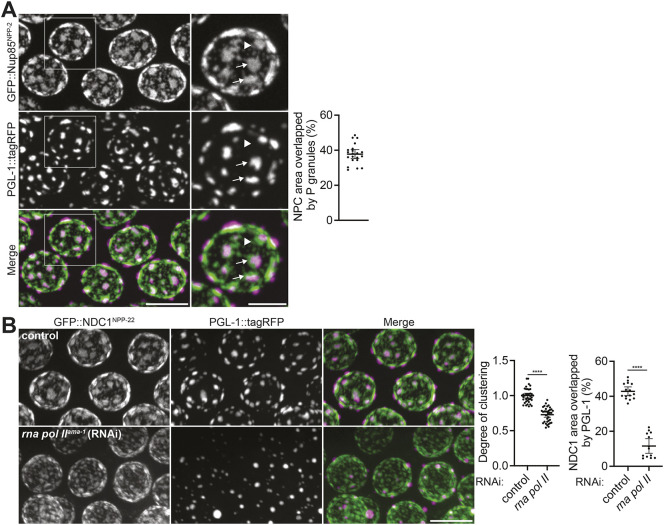
**Nuclear pore clusters associate with P granules and require RNA-polymerase II activity.** (A) Left: Representative confocal micrographs of nuclei co-expressing GFP::Nup85^NPP-2^ and the P granule marker PGL-1::tagRFP. Areas indicated by white boxes are magnified on the right. Arrows denote nuclear pore clusters that extend beyond P granules; arrowhead designates an individual pore that is not covered by a P granule. Right: Quantification of the area occupied by Nup85^NPP-2^ that is overlapped by PGL-1. Error bars represent 95% CI for *n*=21 nuclei. (B) Left: Representative confocal micrographs of nuclei co-expressing GFP::NDC1^NPP-22^ and PGL-1::tagRFP in control germlines or following RNAi to deplete AMA-1. Middle: Normalized quantification of the degree of pore clustering under each condition. Error bars represent 95% CI for *n*≥35 nuclei. Right: Quantification of the total area occupied by NDC1^NPP-22^ that is overlapped by PGL-1. Error bars represent 95% CI for *n*≥13 nuclei. All images are maximum intensity projections. ****P<0.0001 (unpaired *t*-test). Scale bars: 2.5 µm (A, right); 5 µm (A, left; B).

Previous studies have reported that nuclear pore clusters are overlaid by P granules (Sheth et al., 2010; Pitt et al., 2000). In agreement, we found that 100% of pore clusters in pachytene germ cells greater than >1.5 µm^2^ were overlaid by a P granule (*n=*100; Fig. 2A). The overlap between pore clusters and P granules was often imperfect, with nuclear pore clusters extending beyond the periphery of P granules. As previously reported (Sheth et al., 2010), smaller pore clusters and individual pores were not associated with P granules. By comparing the area occupied by P granules versus nuclear pores, we estimate that only ∼40-50% of pores are directly overlaid by a P granule in pachytene nuclei. Overlay of pore clusters by P granules was also observed in embryos in the germline founder cell and its descendants (Fig. S2A).

Previous studies have described a spatial relationship between nuclear pores and nucleoli or the nuclear lamina (Aitchison et al., 1995; Maeshima et al., 2006; Kittisopikul et al., 2021; Wang et al., 2016; Daigle et al., 2001; Sheth et al., 2010; Varberg et al., 2022). However, in contrast to P granules, we observed no correlation between nuclear pore clusters and nucleoli or the nuclear lamina (Fig. S2B,C). Additionally, in agreement with prior studies (Pitt et al., 2000; Schisa et al., 2001), we observed a negative correlation between large nuclear pore clusters and dense chromatin (Fig. S2D).

The dynamics of pore clustering during germline development correlate with transcription, which is highest at the pachytene stage (Gibert et al., 1984; Starck, 1977; Starck et al., 1983; Schisa et al., 2001) and lowest in oocytes (Starck et al., 1983; Belew et al., 2023; Walker et al., 2007). Transcription by RNA polymerase II has been reported to promote perinuclear localization of P granules (Uebel et al., 2020; Sheth et al., 2010; Chen et al., 2020). To examine whether RNA polymerase II activity is also required for nuclear pore clustering, we depleted the large subunit of RNA polymerase II (*ama-1*) by RNA-mediated interference (RNAi). We confirmed a reduction in perinuclear P granules and a reduction in pore clustering in the pachytene region of animals exposed to *ama-1* RNAi (Fig. 2B, Fig. S2E). We conclude that nuclear pore clustering correlates with perinuclear P granules and that both require transcription.

### Pore clustering is reduced in mutants in which P granule association with nuclear pores is weakened

**Fig. 3.**
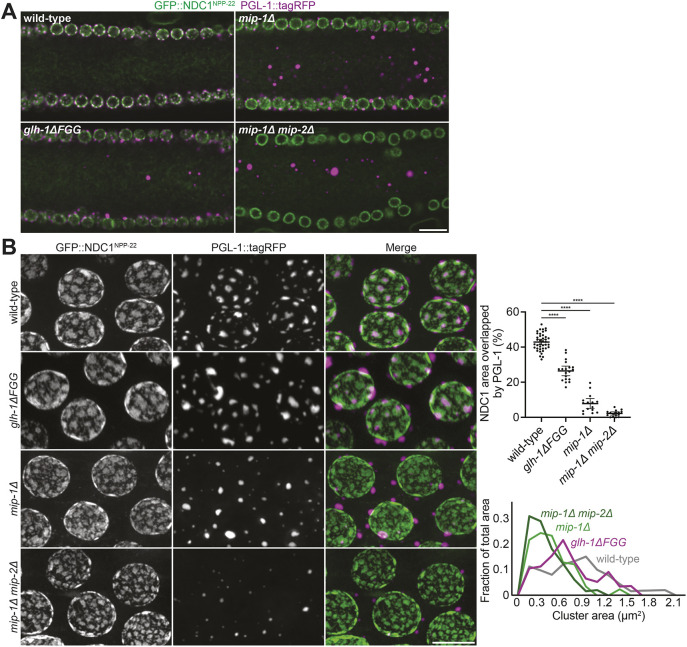
**The P granule proteins MIP-1 and GLH-1 contribute to nuclear pore clustering.** (A) Representative confocal micrographs of cross-sections of pachytene germlines expressing GFP::NDC1^NPP-22^ and the P granule marker PGL-1::tagRFP comparing different genotypes as indicated. (B) Left: Representative confocal micrographs of pachytene nuclei expressing GFP::NDC1^NPP-22^ and PGL-1::tagRFP in germlines of the indicated genotypes. Top right: Quantification of the area occupied by NDC1^NPP-22^ that is overlapped by PGL-1. Error bars represent 95% CI for *n*≥15 nuclei. Bottom right: Frequency graph plotting the size distribution of nuclear pore clusters for each indicated mutant, binned by cluster area (generated from *n*≥147 clusters). Images in A are single focal planes; images in B are maximum intensity projections. ****P<0.0001 (one-way ANOVA). Scale bars: 10 µm (A); 5 µm (B).

The correlation between nuclear pore clusters and P granules raises the possibility that P granules influence pore clustering. Prior studies have implicated the Vasa class helicase GLH-1 and its binding partner, the LOTUS domain protein MIP-1, in the perinuclear localization of P granules (Price et al., 2021, 2023; Cipriani et al., 2021; Chen et al., 2020; Marnik et al., 2019). GLH-1 is one of three related paralogs that contain FG repeats, also found in cytoplasmic facing FG-Nups, leading to a model in which GLH-1 FG repeats mediate association with nuclear pores (Updike et al., 2011). MIP-1 (and its paralog MIP-2) binds to GLH-1 and is required for enrichment of GLH-1 to P granules (Price et al., 2021, 2023; Cipriani et al., 2021). Consistent with these studies, we observed P granules in the cytoplasm away from nuclei in the mitotic and pachytene regions of *glh-1*Δ*FGG* mutants lacking FG repeats in GLH-1, in *mip-1*Δ single mutants and in *mip-1*Δ *mip-2*Δ double mutants (Fig. 3A, Fig. S3A). Coverage of nuclear pores by P granules was reduced in all three mutants, although to different extents with *mip-1*Δ *mip-2*Δ double mutants most severely affected (Fig. 3B, Fig. S3B). Nuclear pore clustering was also reduced in the mutants, in a manner proportional to the loss of coverage by P granules (Fig. 3B). These observations suggest that perinuclear P granules enhance nuclear pore clustering.

### Cytoplasmic-facing FG-Nups are required for nuclear pore clustering

**Fig. 4.**
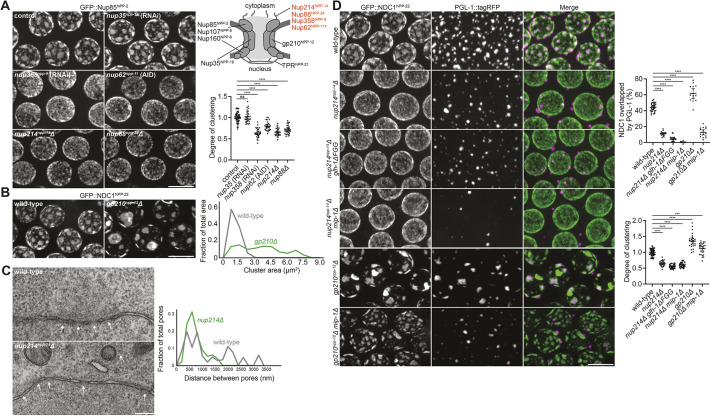
**Cytoplasmic-facing FG-Nups drive germline nuclear pore clustering.** (A) Left: Representative confocal micrographs of pachytene nuclei expressing GFP::Nup85^NPP-2^ following RNAi-mediated depletion of Nup358^NPP-9^ or Nup35^NPP-19^, auxin-mediated degradation of Nup62^NPP-11^, or in *nup214^npp-14^*Δ or *nup88^npp-24^*Δ mutants. Top right: Schematic depicting the nuclear pore complex architecture, with the location of Nups tested for their role in nuclear pore clustering. Nups denoted in orange were found to contribute to clustering of germline nuclear pores. Asterisk indicates that Nup62^NPP-11^ localizes to both the central channel and the cytoplasmic face of the pore. Bottom right: Normalized quantification of the degree of pore clustering under each condition. Error bars represent 95% CI for *n*≥27 nuclei. (B) Left: Representative confocal micrographs of pachytene nuclei expressing GFP::NDC1^NPP-22^ in wild-type versus *gp210^npp-12^*Δ mutant germlines. Right: Frequency graph of the size distribution of nuclear pore clusters, binned by cluster area (generated from *n*≥291 clusters). (C) Left: Representative electron micrographs of nuclear pores in the pachytene germline of wild type versus *nup214^npp-14^*Δ mutants. White arrows indicate nuclear pores. Right: Frequency graph of the distribution of distance between adjacent nuclear pores, binned by distance (generated from *n*≥83 pores). (D) Left: Representative confocal micrographs of pachytene nuclei expressing GFP::NDC1^NPP-22^ and the P granule marker PGL-1::tagRFP in wild type versus the indicated mutants. Top right: Quantification of the area occupied by NDC1^NPP-22^ that is overlapped by PGL-1. Error bars represent 95% CI for *n*≥14 nuclei. Bottom right: Normalized quantification of the degree of pore clustering under each indicated condition. Error bars represent 95% CI for *n*≥26 nuclei. All confocal micrographs are maximum intensity projections. ***P<0.001; ****P<0.0001 (one-way ANOVA). ns, not significant. Scale bars: 200 nm (C); 5 µm (A,B,D).

Aberrant clustering of nuclear pores has been reported in various Nup mutants (Aitchison et al., 1995; Cohen et al., 2003; Galy et al., 2003; Ródenas et al., 2009; Heath et al., 1995; Pemberton et al., 1995; Xie et al., 2016; Kittisopikul et al., 2021; Guo and Zheng, 2015; Liu et al., 2000). To test whether specific Nups influence pore clustering in the *C. elegans* germline, we systematically depleted a set of ten Nups representing all major Nup complexes using a combination of CRISPR deletion mutants, RNAi, and auxin-induced degradation (Ashley et al., 2021). Depletion of core and nucleoplasmic Nups had no detectable effect on clustering (Fig. 4A, Fig. S4A), with the exception of the Y-complex component Nup160 (NPP-6) and the transmembrane Nup gp210 (NPP-12), depletion of which led to increased pore clustering (Fig. 4B, Fig. S4A) as previously reported (Heath et al., 1995; Cohen et al., 2003). In contrast, loss of cytoplasmic-facing FG-Nups [Nup62 (NPP-11), Nup358 (NPP-9), Nup214] and the Nup214 binding partner Nup88 (NPP-24) dramatically reduced clustering, causing a re-distribution of pores across pachytene nuclei (Fig. 4A). We confirmed a reduction in pore clustering in *nup214*Δ mutants using four additional Nup markers, as well as electron microscopy to visualize individual pores directly (Fig. 4C, Fig. S4B). The *nup214*Δ mutant also reduced pore clustering in embryonic germ cells (Fig. S4C). Surprisingly, *nup214*Δ mutants maintained normal levels of clustering in the mitotic region of the germline (Fig. S4D). We conclude that FG-Nups promote nuclear pore clustering in the pachytene germline and early embryos.

### Nuclear pore and P granule clustering are interdependent

In *nup214*Δ mutants, P granule association with nuclei was reduced in the pachytene region but had a wild-type appearance in the mitotic zone, where *nup214*Δ mutants maintained normal pore clustering (Fig. 4D, Fig. S4E). P granule association with nuclei was also reduced following depletion of Nup62 or Nup358, two independent perturbations that reduce pore clustering (Fig. S4F). These observations raise the possibility that clustering enhances P granule association with pores. In agreement, we observed a larger proportion of pores associated with P granules in the hyper-clustering *gp210*Δ mutant (Fig. 4D)*.* Combining *glh-1*Δ*FGG* and/or *mip-1*Δ with *nup214*Δ or *gp210*Δ mutants reduced both P granule association with nuclei and pore clustering in the pachytene germline (Fig. 4D)*.* The majority of nuclear pore clusters that remained in *gp210*Δ *mip-1*Δ double mutants were not associated with P granules, indicating that, although P granules enhance clustering, they are not essential for clustering.

In *C. elegans* oocytes, in which P granules become cytoplasmic, FG-Nups form cytoplasmic condensates that associate with the surface of P granules (Fig. S4G) (Pitt et al., 2000; Sheth et al., 2010; Jud et al., 2007; Patterson et al., 2011; Thomas et al., 2023). FG-Nup condensation was reduced following depletion of *mip-1* and *mip-2* by RNAi, which reduces P granules in oocytes (Fig. S4H) (Cipriani et al., 2021), suggesting that the surface of P granules stimulates FG-Nup condensation. We conclude that nuclear pore clustering and P granule association with nuclear pores are processes that reinforce each other.

### Loss of pore clustering and perinuclear P granules disrupts the localization of other nuage condensates

**Fig. 5.**
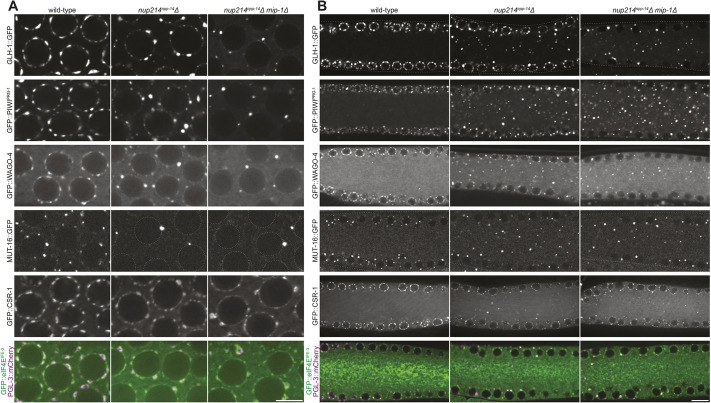
**Nuclear pore clustering is required for perinuclear nuage condensates.** (A) Representative confocal micrographs of pachytene nuclei expressing the P granule markers GLH-1::GFP, GFP::PIWI^PRG-1^, and PGL-3::mCherry, the Z granule marker GFP::WAGO-4, the Mutator foci marker MUT-16::GFP, the D and P granule marker GFP::CSR-1, or the P-body marker GFP::eIF4E^IFE-3^ in wild-type, *nup214^npp-14^*Δ and *nup214^npp-14^*Δ *mip-1*Δ mutant germlines. (B) Representative confocal micrographs of cross-sections of pachytene regions expressing same markers as in A in wild-type, *nup214^npp-14^*Δ, and *nup214^npp-14^*Δ *mip-1*Δ mutant germlines. All images are single focal planes. Scale bars: 5 µm (A); 10 µm (B).

In addition to P granules, several other condensate types assemble around nuclei in pachytene germ cells, including Z granules, Mutator foci, germline P-bodies, SIMR foci, D granules and E granules (Phillips et al., 2012; Wan et al., 2018; Manage et al., 2020; Noble et al., 2008; Huang et al., 2024; Du et al., 2023). To examine the effect of loss of pore clustering and perinuclear P granules on other nuage compartments, we systematically compared the distribution of seven endogenously tagged nuage proteins in wild-type, *nup214*Δ and *nup214*Δ *mip-1*Δ adult hermaphrodites. We found that, as expected, the P granule proteins GLH-1, PRG-1 and PGL-3 accumulated in granules that were depleted from the nuclear periphery (Fig. 5A) and enriched in the cytoplasm (Fig. 5B) in *nup214*Δ and *nup214*Δ *mip-1*Δ mutants, with the double mutant exhibiting a more severe depletion of perinuclear granules (Fig. S5A). The Z granule marker WAGO-4 and the Mutator foci marker MUT-16 behaved similarly, except that MUT-16 appeared equally affected in *nup214*Δ and *nup214*Δ *mip-1*Δ mutants (Fig. 5) and was less affected by the *mip-1*Δ mutation alone (Fig. S5B). The Argonaute CSR-1 has been recently described as a marker for both P and D granules, as it partially overlaps with the D granule marker DDX19 (DDX-19) and remains perinuclear in a *mip-1*Δ mutant (Huang et al., 2024). We found that CSR-1 localized to cytoplasmic granules (likely P granules) in *nup214*Δ and *nup214*Δ *mip-1*Δ mutants and also maintained robust localization at the nuclear periphery (Fig. 5, Fig. S5C). DDX19 behaved similarly (Fig. S5D). The P-body markers eIF4E^IFE-3^ (IFE-3), LSM14 (CAR-1) and DDX6 (CGH-1) localized both at the nuclear periphery and around P granules in the cytoplasm (Fig. 5, Fig. S5E,F). We conclude that loss of pore clustering causes a relocalization of P granules, and all associated condensates tested, to the cytoplasm, with D granules and P-bodies also maintaining a nuclear-associated pool.

Double labeling with PGL-1 revealed that Z granules merged with P granules in *nup214*Δ mutants (Fig. S5G). Mutator foci and P-bodies colocalized with P granules in the cytoplasm of *nup214*Δ mutants but remained distinct condensates that were either next to (MUT-16) or surrounding (eIF4E^IFE-3^) P granules (Fig. S5F). We conclude that localization to the nuclear periphery is not essential for P granules to interact with Mutator foci and P-bodies but is required to prevent P granules from merging with Z granules.

### Perinuclear localization of P granules is not essential for fertility

**Fig. 6.**
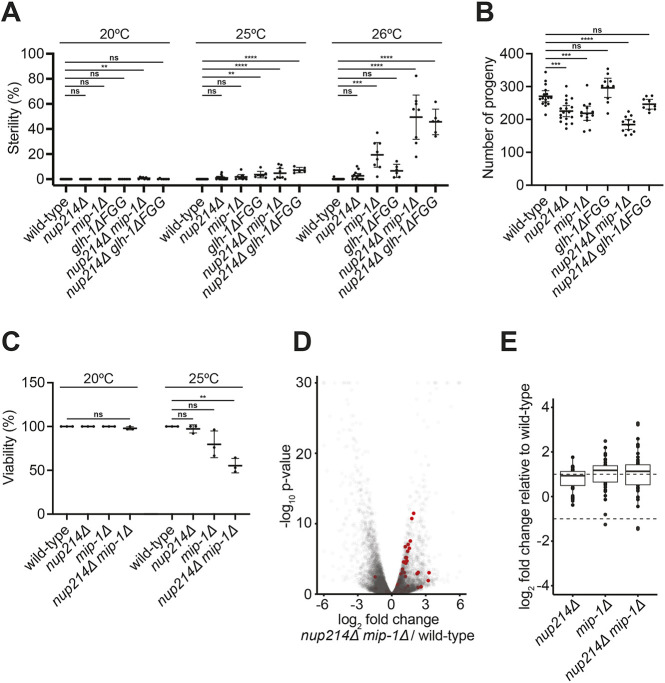
***nup214^npp-14^*Δ and *mip-1*Δ mutants are fertile under standard growth conditions and express elevated levels of histone transcripts.** (A) Quantification of the percentage of sterile hermaphrodites for wild type versus *nup214^npp-14^*Δ, *mip-1*Δ and *glh-1*Δ*FGG* mutants grown at the indicated temperatures for one generation. Error bars represent 95% CI for *n*≥591 animals from *N*=3 independent experiments. (B) Brood sizes of wild type versus the indicated mutants grown at 20°C. Error bars represent 95% CI for *n*≥9 animals from *N*≥2 independent experiments. (C) Embryonic viability of wild type versus *nup214^npp-14^*Δ and *mip-1*Δ mutants from mothers grown at 20°C versus 25°C. Error bars represent 95% CI for *n*≥95 embryos from *N*=3 independent experiments. (D) Volcano plot of fold change in transcript abundance between *nup214^npp-14^*Δ *mip-1*Δ and wild type against −log_10_ adjusted *P*-value. Replication-dependent histone transcripts are highlighted in red. (E) log_2_ fold changes of all replication-dependent histone transcripts with a minimum baseMean of 10 in either *nup214^npp-14^*Δ, *mip-1*Δ or *nup214^npp-14^*Δ *mip-1*Δ mutants relative to wild type. Upper and lower hinges correspond to 25th and 75th percentiles. Whiskers are 1.5× the IQR (inter-quartile range, which is the distance between the 25th and 75th percentiles). Dashed lines denote the log_2_ fold changes used to define significant misregulation. **P<0.01; ***P<0.001; ****P<0.0001 (one-way ANOVA). ns, not significant.

Compared to nearly 50% in wild type, only 11%, 26%, 8%, 5% and 0.5% of nuclear pore-rich areas in pachytene nuclei were covered by P granules in *nup214*Δ, *glh-1*Δ*FGG*, *mip-1*Δ, *nup214*Δ *glh-1*Δ*FGG*, and *nup214*Δ *mip-1*Δ mutants, respectively. Remarkably, these genotypes were nearly 100% fertile at 20°C (Fig. 6A). For a subset of mutants, we observed low levels of sterility at 25°C, which increased at 26°C. We also observed small reductions in brood sizes in *nup214*Δ and *mip-1*Δ mutants but not *glh-1*Δ*FGG* mutants (Fig. 6B) as well as reduced embryonic viability of *mip-1*Δ and *nup214*Δ *mip-1*Δ mutants at elevated temperatures (Fig. 6C). We conclude that perinuclear localization of P granules (as well as Z granules and Mutator foci) is largely dispensable for fertility under standard growth conditions.

### *nup214*Δ *mip-1*Δ mutants accumulate elevated levels of histone transcripts

To examine the consequences of loss of perinuclear P granules at the molecular level, we analyzed by RNAseq the transcriptome of adult hermaphrodites grown at 20°C comparing wild type, *nup214*Δ, *mip-1*Δ, and *nup214*Δ *mip-1*Δ mutants*.* Among the nearly 18,000 transcripts detected in these analyses, we identified only ∼11% of transcripts misregulated by 2-fold or more, and 4% of transcripts misregulated by 4-fold or more, in *nup214*Δ *mip-1*Δ double mutants (Fig. 6D). Gene ontology (GO) term analyses comparing differentially expressed genes in each genotype identified three classes of genes upregulated in all three genotypes (Fig. S6A). Two gene classes, implicated in sperm development and stress responses, have already been reported as upregulated in *mip-1*Δ mutants (Price et al., 2023). Here, we focus on the third group: replication-dependent histones. These transcripts are not polyadenylated and therefore were not recovered in prior analyses of *mip-1*Δ mutants that focused on polyadenylated transcripts. Interestingly, prior studies identified the same three classes of genes as transcripts for which abundance in polysomes increase (stress and replication-dependent histones) or decrease (sperm) in *glh-1* mutants (Rochester et al., 2022).

The *C. elegans* genome contains 64 replication-dependent histone genes coding for the four core histones (H2A, H2B, H3 and H4) (Keall et al., 2007). As a group, histone genes were upregulated in the *nup214*Δ *mip-1*Δ double and *mip-1*Δ single mutants and, more weakly, in the *nup214*Δ single mutant (Fig. 6E). Genes coding for all four histone types were upregulated in the *nup214*Δ *mip-1*Δ mutants (Fig. S6B). Replication-dependent histone transcripts are targeted by antisense sRNAs (Reed et al., 2020; Barucci et al., 2020). *C. elegans* accumulate several classes of sRNAs, distinguishable by the types of Argonautes to which they are bound (Sundby et al., 2021). We sequenced sRNAs and observed similar levels of sRNAs mapping to target genes for each Argonaute class in *nup214*Δ *mip-1*Δ animals compared to wild type (Fig. S6C). sRNAs antisense to histone transcripts, however, were reduced in *nup214*Δ *mip-1*Δ mutants (Fig. S6B,D). Genome browser views of two histone clusters confirmed the increase in mRNA and decrease in sRNAs at histone loci in *nup214*Δ *mip-1*Δ mutants (Fig. S6E). We conclude that *nup214*Δ *mip-1*Δ, *mip-1*Δ, and to a lesser extent *nup214*Δ, mutants accumulate excess replication-dependent histone transcripts and that this overexpression correlates in *nup214*Δ *mip-1*Δ mutants with reduced sRNAs at the same loci.

### Excess histone transcripts accumulate in P granules and in the rachis of mutants with reduced perinuclear P granules

To determine where histone transcripts accumulate in the mutants, we used fluorescence *in situ* hybridization (FISH) to visualize histone transcripts. We used four probes, one for each histone type (the high sequence conservation among genes of the same histone type means that each probe is predicted to capture transcripts from all genes in that class). In dividing cells, replication-dependent histone genes are transcribed during S phase and degraded post-S phase (Marzluff and Koreski, 2017). In the *C. elegans* germline, histone transcripts are also transcribed post-S phase in pachytene germ cells and stored as maternal transcripts in oocytes for transmission to embryos (Gleason et al., 2023; Han et al., 2019; Boeck et al., 2016). As expected, in adult hermaphrodites, we detected histone transcripts only in the germline and not in somatic tissues, which have ceased proliferation (Fig. S7A) (Keall et al., 2007). We detected histone transcripts around groups of nuclei in the mitotic germline where germ cells are dividing asynchronously (Fig. S7B), and throughout the shared cytoplasm in the pachytene region (Fig. S7A).

**Fig. 7.**
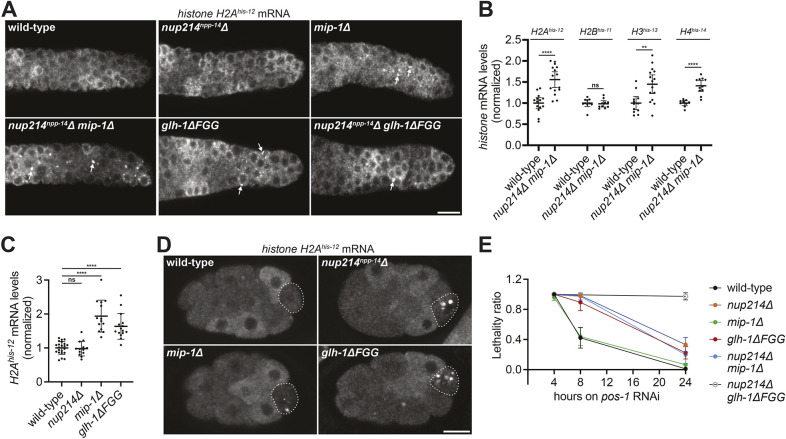
**Mutants with impaired P granule nuclear association accumulate histone transcripts in P granules and are RNAi defective.** (A) Representative confocal micrographs of mitotic regions of the indicated genotypes hybridized to an antisense probe against *histone H2A^his-12^* mRNA. White arrows denote accumulation of *histone H2A^his-12^* mRNA in granules. (B) Quantification of the levels of each indicated histone transcript in the pachytene rachis of wild type versus *nup214^npp-14^*Δ *mip-1*Δ mutants. Quantification was performed using the images shown in Fig. S7D. Error bars represent 95% CI for *n*≥9 germlines. (C) Quantification of the levels of *H2A^his-12^* mRNA in the pachytene rachis of wild type versus the indicated mutants. Error bars represent 95% CI for *n*≥10 germlines. (D) Representative confocal micrographs of 16-cell-stage embryos of the indicated genotypes hybridized to an antisense probe against histone *H2A^his-12^* mRNA. Dashed lines denote the P cell. (E) Graph plotting the lethality ratio among F1 progeny derived from wild type versus the indicated mutants fed *pos-1* RNAi for the indicated amount of time. Error bars represent 95% CI for *n*≥114 embryos from *N*=3 independent experiments. All images are single focal planes. **P<0.01; ****P<0.0001 [unpaired *t*-test (B) or one-way ANOVA (C)]. ns, not significant. Scale bars: 10 µm.

We observed a similar distribution of histone transcripts in *nup214*Δ *mip-1*Δ animals except that histone transcripts accumulated in P granules specifically in the mitotic zone (Fig. 7A, Fig. S7C). H2A, H3 and H4 transcript levels were also elevated in the pachytene cytoplasm compared to wild type (Fig. 7B, Fig. S7D). We also observed granule accumulation in the mitotic zone and increased levels in the pachytene cytoplasm in *mip-1*Δ and *glh-1*Δ*FGG* single mutants, but not in *nup214*Δ mutants (Fig. 7A,C).

Nuclear pore clustering is not affected in the mitotic germline of *nup214*Δ mutants but is affected in embryos. To examine whether histone transcripts are misregulated in embryos, we characterized the distribution of *H2A^his-12^* transcripts in embryos. In wild type, as expected, we observed low, uniform levels of maternal histone transcripts in 1- to 4-cell-stage embryos before the onset of zygotic transcription (Fig. S7E). Starting at the 8-cell stage, we observed higher levels of histone transcripts in a subset of cells, consistent with S-phase synthesis and post-S-phase degradation in blastomeres, which divide asynchronously at this stage. We observed cycling histone transcripts in all lineages after the 8-cell stage, including the P lineage (P_3_ and P_4_). Remarkably, in *nup214*Δ, *mip-1*Δ and *glh-1*Δ*FGG* mutant embryos, the distribution of *H2A^his-12^* transcripts was identical to that of wild type, except that *H2A^his-12^* accumulated transiently in P granules specifically in P_3_ and P_4_ (Fig. 7D). In contrast, histone transcripts rarely accumulated in P granules in wild-type embryos, which rapidly turn on and degrade histone transcripts in P_3_ and P_4_ as in somatic blastomeres. Combining these observations with our observations in adult germlines, we conclude that P granules defective in nuclear pore attachment accumulate histone transcripts specifically in cycling cells that are transcribing and turning over histone transcripts.

### Mutants with reduced perinuclear P granules are RNAi defective

The ectopic accumulation of histone transcripts in mutants with reduced perinuclear P granules led us to explore whether these mutants might also be defective in degrading mRNAs targeted by RNAi. To examine this possibility, we tested each mutant for sensitivity to RNAi against *pos-1*, an essential maternal transcript transcribed in the pachytene germline (Tabara et al., 1999). Whereas wild-type animals produced 100% dead progeny within ∼24 h of *pos-1* RNAi, we observed partial to complete RNAi resistance in mutants in which P granule association with nuclei is disrupted (Fig. 7E).

To confirm that this effect was due to a failure to turn over the targeted mRNA, we used FISH to monitor levels of *mex-6*, a maternal nonessential transcript (Schubert et al., 2000), in animals exposed to *mex-6* RNAi. Following 24 h of *mex-6* RNAi, *mex-6* transcripts were severely reduced in wild-type germlines (Fig. S7F). In contrast, *mex-6* mRNA levels were only partially reduced in the pachytene region of *nup214*Δ *mip-1*Δ and *nup214*Δ *glh-1*Δ*FGG* mutants. We conclude that degradation of messages targeted by RNAi is less effective in *nup214*Δ *mip-1*Δ and *nup214*Δ *glh-1*Δ*FGG* animals.

## DISCUSSION

In this study, we examine the mechanisms underlying nuclear pore clustering in the *C. elegans* germline. We identify cytoplasmic-facing FG-Nups as the main drivers of pore clustering, leading to a model in which cytoplasmic FG-Nups interact between neighboring nuclear pores to form a cohesive network (Fig. S8). We suggest that nuclear pore clusters form favorable binding surfaces for P granules, which contain FG repeat proteins that reinforce pore clustering. We demonstrate that P granule coverage of nuclear pores is reduced to less than 1% in mutants lacking the non-essential FG-Nup Nup214 and the P granule protein MIP-1. Remarkably *nup214*Δ *mip-1*Δ mutants are viable and largely fertile and exhibit misregulation of only a small number of transcripts, including replication-dependent histone transcripts. We propose a model whereby close association with nuclear pores accelerates RNA dynamics in and out of P granules, which tunes their levels in the germline.

### Nuclear pore clustering requires cytoplasmic-facing FG-Nups and is reinforced by P granules

Systematic depletion of ten Nups, representative of every nuclear pore subcomplex, revealed that cytoplasmic-facing FG-Nups are specifically required to promote pore clustering in the germline. Strikingly, the FG-Nups required for pore clustering (Nup214, Nup358, Nup62 and Nup88) are also required for the assembly of non-essential cytoplasmic Nup condensates in *C. elegans* oocytes (Thomas et al., 2023). These findings suggest that the cohesive interactions that drive Nup condensation in the cytoplasm also facilitate pore clustering at the nuclear envelope by linking adjacent pores. In support of this model, electron microscopy studies have detected electron-dense regions linking clusters of pores at the base of P granules, which may correspond to a cytoplasmic layer of cohesive FG-Nups (Pitt et al., 2000; Sheth et al., 2010; Schisa et al., 2001). Additionally, composite structures and molecular dynamics simulations predict that FG-Nups extend a significant distance from the cytoplasmic face of the pore (Bley et al., 2022; Yu et al., 2023), and could therefore participate in interaction networks that extend beyond individual pores. Finally, Nup98, a highly cohesive FG-Nup, has been proposed to act as molecular ‘Velcro’ connecting multiple pore subunits in a multivalent network (Onischenko et al., 2017). In *C. elegans*, Nup98 is required for robust Nup condensation in oocytes and is reported to promote P granule association with nuclei (Price et al., 2021; Voronina and Seydoux, 2010; Updike and Strome, 2009; Thomas et al., 2023). Nup98 is essential for pore complex assembly (Onischenko et al., 2017; McCloskey et al., 2018), which precluded our ability to test for a possible role in pore clustering.

Several lines of evidence suggest that P granules reinforce nuclear pore clustering. P granules are unique to the germline and their perinuclear localization correlates closely with pore clustering. P granules begin as small condensates in the distal mitotic region and increase in size as germ cells enter meiosis (Pitt et al., 2000); correspondingly, the size of pore clusters increases as germ cells transition from the mitotic region to pachytene. P granules detach from nuclei in oocytes and nuclear pores return to a more homogeneous distribution (Strome and Wood, 1982; Updike and Strome, 2010). P granules reassociate progressively with the nuclear envelope in embryonic germline precursors and nuclear pore clusters reform at this stage. Pore clustering is reduced by mutations in specific P granule proteins, including the RNA-dependent RNA polymerase EGO-1 (Vought et al., 2005), the P granule scaffold MIP-1, and the FG repeat-containing helicase GLH-1. Most strikingly, deletion of the FG repeats in GLH-1 is sufficient to reduce P granule attachment to nuclei and pore clustering, and both are reduced further by the additional loss of the FG-Nup Nup214. Studies have shown that GLH-1 functions redundantly with GLH-2 and GLH-4, which also have FG repeats (Spike et al., 2008; Marnik et al., 2019; Chen et al., 2020); redundancy may explain why the *glh-1*Δ*FGG* mutation has a comparatively mild effect on P granule nuclear association. These observations suggest that cohesive FG–FG interactions create a network at the nuclear surface that drives the co-clustering of nuclear pores and P granules. Consistent with this model, when P granules become cytoplasmic in oocytes, Nup clusters dissipate at the nuclear surface and cytoplasmic FG-Nups condense on the surface of P granules (this study; Patterson et al., 2011; Pitt et al., 2000; Thomas et al., 2023; Sheth et al., 2010).

### Nascent RNA likely facilitates nuclear pore/P granule co-clustering

Several lines of evidence suggest that nascent transcripts emerging from nuclear pores enhance pore association with P granules. First, the extent of P granule and pore co-clustering correlates with changes in transcription activity during germline development. Transcription peaks in the pachytene germline (Gibert et al., 1984; Starck, 1977; Starck et al., 1983; Schisa et al., 2001) where nuclear pore/P granule co-clusters are largest, and is silenced as oocytes enter diakinesis, during which pore clusters dissipate and P granules become cytoplasmic (Starck et al., 1983; Belew et al., 2023; Walker et al., 2007). Resumption of transcription in the embryonic germline correlates with pore clustering and re-association of P granules with nuclear pores (Seydoux and Dunn, 1997). Perinuclear germ granules in *Drosophila* and mice have also been correlated with periods of active transcription (Kibanov et al., 2011; Meikar et al., 2011). Together, these observations suggest that nascent transcripts drive the formation of perinuclear granules. Consistent with this view, we find that loss of RNA polymerase II reduces pore clustering and also releases P granules into the cytoplasm as shown in prior studies (Sheth et al., 2010; Uebel et al., 2020; Chen et al., 2020).

RNA is a common component of biomolecular condensates and emerging research suggests that RNA plays a key role in condensate assembly and structure (Van Treeck and Parker, 2018). RNA promotes the phase separation of RNA-binding proteins *in vitro* (Patel et al., 2015; Lin et al., 2015; Molliex et al., 2015) and *in vivo* (Cabral et al., 2022; Putnam et al., 2023). Interestingly, a systematic survey showed that Nup358 and the Nup214-Nup88-Nup98 subcomplex exhibit some affinity for RNA (Bley et al., 2022). Given the high levels of transcription in the pachytene germline, it is possible that nascent transcripts enhance FG repeat condensation by helping to bridge FG repeats between neighboring pores and P granules.

### Loss of P granules from the nuclear periphery disrupts the layered organization of nuage

Throughout the pachytene region, nuage condensates arrange at the nuclear periphery in reproducible layers with D granules closest to the nuclear surface, followed by P granules, Z granules and Mutator foci, and finally germline P-bodies facing the cytoplasm. This organization has been proposed to facilitate the transfer of nascent mRNAs and sRNAs from one condensate to another (Sundby et al., 2021; Phillips and Updike, 2022; Ouyang and Seydoux, 2022). Our findings indicate that loss of pore clustering and perinuclear P granules leads to a global reorganization of nuage condensates: Mutator foci and Z granules leave the nuclear periphery and relocalize with P granules in the cytoplasm, and D granules and P-bodies become split between perinuclear pools and peri-P granule pools in the cytoplasm. Interestingly, P and Z granules mix into hybrid condensates in our mutants, as also observed in wild-type oocytes where P granules naturally detach from nuclei (Wan et al., 2018). These observations suggest that de-mixing of P granules and Z granules requires interactions between P granule components and FG-Nups and/or nascent RNAs exiting from nuclear pores. Our findings also suggest that nuclear factors other than FG-Nups drive the perinuclear localization of D granules and P-bodies. Together, these observations suggest that the layered organization of nuage is not governed by a strict linear hierarchy of affinities between each condensate type and the nuclear periphery. Rather, nuage organization is an emergent property that arises from a complex web of condensate–condensate interactions and condensate–nuclear periphery interactions, involving FG-Nups and likely other nuclear factors.

### The perinuclear localization of P granules is not essential for fertility

Prior studies estimated that ∼75% of nuclear pores were covered by P granules, leading to a model in which most exported transcripts are routed to P granules for surveillance (Pitt et al., 2000; Sheth et al., 2010). Those studies, however, were performed before the identification of other nuage compartments and availability of high-resolution microscopic methods. From our imaging of endogenously tagged proteins, we estimate that only ∼40-50% of germline pores are directly overlaid by a P granule. In this respect, it is perhaps not surprising that mutants in which the connection between P granules and pores is disrupted are fertile and exhibit misregulation of only a minority of genes at 20°C, the optimal growth temperature for *C. elegans*. This stands in contrast to mutations in P granule proteins, which lead to immediate sterility or sterility after a few generations (Kawasaki et al., 1998, 2004; Spike et al., 2008; Simon et al., 2014). Our observations suggest that P granule proteins can still regulate transcripts after they have entered the cytoplasm. P granule proteins could recruit transcripts in the granules from the cytoplasm or could function in the cytoplasm themselves. A recent survey found that most nuage proteins in *C. elegans* also localize to the cytoplasm (Huang et al., 2024).

Although the mutants we tested were fertile at temperatures up to 25°C, a percentage of *mip-1*Δ, *nup214*Δ *mip-1*Δ and *nup214*Δ *glh-1*Δ*FGG* mutants became sterile at 26°C, a stressful temperature outside the optimal temperature range of 15-25°C (Kyriakou et al., 2022). Decreased fertility at elevated temperatures is commonly observed in germ granule protein mutants (Chen et al., 2020; Marnik et al., 2019; Cipriani et al., 2021; Gallo et al., 2010; Kawasaki et al., 1998, 2004; Spike et al., 2008; Rogers and Phillips, 2020). The penetrance of fertility defects observed at high temperature were specific to each mutant, raising the possibility that the underlying causes are gene specific and not necessarily linked to loss of perinuclear P granules.

### Hypothesis: Close association with pores accelerates RNA flux through P granules

The most striking phenotype shared by all mutants with P granules detached from pores we examined (*nup214*Δ, *mip-1*Δ, *glh-1*Δ*FGG* and *nup214*Δ *mip-1*Δ mutants) was an accumulation of histone transcripts in P granules in cycling germ cells. Replication-dependent histone transcripts lack introns and polyadenylated tails and require recognition of a 3′ UTR stem-loop for nuclear export, translation during S phase and degradation post-S phase (Keall et al., 2007; Marzluff et al., 2008). In *C. elegans*, the Argonaute CSR-1 is required for proper processing of histone transcripts downstream of the stem loop (Avgousti et al., 2012). Depletion of CSR-1 by RNAi in wild-type worms, however, was not sufficient to lead to histone mRNA accumulation in P granules, and did not suppress P granule accumulation in mutants, ruling out a defect in CSR-1 function as the sole cause for this phenotype (Fig. S7G). Loss of CSR-1 (and its co-factors EGO-1, EKL-1 and DRH-3) leads to enlarged P granules at the nuclear periphery and in the cytoplasm (Andralojc et al., 2017). It will be important in the future to investigate how *csr-1* phenotypes relate to the *nup214*Δ *mip-1*Δ phenotypes reported here.

In wild-type animals, histone transcripts are naturally targeted by antisense sRNAs, possibly because histone mRNA decay intermediates produced during S phase are occasionally recognized by RNA-dependent RNA polymerases that synthesize antisense sRNAs. Consistent with this hypothesis, *prg-1* mutants, which lack PRG-1/piRNA complexes that engage most of the sRNA amplification machinery, accumulate excess anti-histone sRNAs and reduced levels of histone transcripts (Reed et al., 2020; Barucci et al., 2020; Spichal et al., 2021). We observed the opposite phenotype in *nup214*Δ *mip-1*Δ mutants: lower levels of anti-histone sRNAs and higher levels of histone transcripts. The primary cause leading to this phenotype is not clear. One possibility is that detachment from nuclear pores lowers the overall flux of mRNA molecules entering and exiting the P granules. The lower flux could trap histone transcripts in the granules away from degradative enzymes in the cytoplasm, with the net effect of slowing down histone mRNA decay and sRNA amplification (Fig. S8). Consistent with a general defect in mRNA flux through P granules, we also observed delayed turnover of transcripts targeted by RNAi, which has also been correlated with transient transcript accumulation in granules (Ouyang et al., 2022). In this model, close association with pores serves primarily to maintain rapid, directional mRNA flow between P granules, other nuage condensates and/or the cytoplasm, possibly to fine-tune the balance between Argonaute recognition, sRNA amplification and RNA decay, which could occur in different nuage layers (Fig. S8). Evaluation of this and other models will require methods that allow direct examination of RNA dynamics *in vivo*. Although the significance of the perinuclear organization of nuage condensates remains mysterious, our findings indicate that obligatory passage through a P granule before accessing the cytoplasm is not a prerequisite for the regulation of most germline transcripts.

## MATERIALS AND METHODS

### *C. elegans* strains and culture

*C. elegans* were cultured using standard methods (Brenner, 1974). Briefly, worms were maintained at 20°C on normal nematode growth media (NNGM) plates (IPM Scientific Inc., 11006-548) seeded with OP50 bacteria. For all experiments, worms were synchronized as day 1 adults using vulval morphology to stage L4 larvae. Endogenous edits were performed using CRISPR/Cas9-mediated genome editing as described previously (Paix et al., 2017). Strains with a PHX strain designation were generated by SunyBiotech. Standard crosses were used to generate strains with multiple genomic edits. All strains used or generated in this study are described in Table S1. Strains have been deposited at the *Caenorhabditis* Genetics Center (CGC) or are available upon request.

### RNAi

RNAi was performed by feeding (Timmons and Fire, 1998). RNAi vectors were obtained from the Ahringer or Open Biosystems libraries and sequence verified, or alternatively cloned from *C. elegans* cDNA and inserted into the T777T enhanced RNAi vector (Addgene plasmid #113082). RNAi feeding vectors were transformed into HT115 bacteria, grown to log phase in LB broth+100 μg/ml ampicillin at 37°C, induced with 5 mM IPTG for 45 min, and plated on RNAi plates (50 μg/ml carbenicillin, 1 mM IPTG; IPM Scientific Inc., 11006-529), and allowed to dry overnight at room temperature. For depletion of RNA polymerase II^AMA-1^ (Fig. 2B, Fig. S2E), RNAi feeding was performed with L4 larvae for 30 h at 20°C. For *pos-1* RNAi (Fig. 7E), L4 larvae were fed RNAi at 20°C for the indicated amount of time. For all other experiments, RNAi feeding was performed with L4 larvae for 18-24 h at 20°C. For all experiments, control animals were fed HT115 bacteria transformed with empty vector.

### Immunofluorescence

For immunostaining of embryos, gravid adults were placed into 7 μl of L-15 media (Gibco, 21083-027) on a poly-L-lysine-coated slide and compressed with a coverslip to extrude embryos. For immunostaining of germlines, adults were dissected on poly-L-lysine slides to extrude the germline, and a coverslip was placed gently on top. In both cases, slides were immediately frozen on aluminum blocks pre-chilled with dry ice. After >5 min, coverslips were removed to permeabilize embryos (freeze-cracking), and slides were fixed for at least 24 h in pre-chilled methanol at −20°C. Slides were then incubated in pre-chilled acetone for 10 min at −20°C, and blocked in PBS-Triton (PBS, 0.1% Triton X-100, 0.1% bovine serum albumin) for at least 30 min at room temperature. Slides were then incubated overnight in primary antibody in a humid chamber at 4°C. Slides were washed three times (10 min each wash) in PBS-Triton at room temperature, incubated in secondary antibody for 2 h in a humid chamber at room temperature, and washed three times (10 min each wash) in PBS-Triton at room temperature. Slides were then washed once in PBS before being mounted using Prolong Glass Antifade Mountant with NucBlue (Thermo Fisher Scientific, P36981). Primary antibodies were as follows: anti-Nup358^NPP-9^ (1:250; Novus Biologicals, 48610002); anti-PGL-1/3 (1:10; Developmental Studies Hybridoma Bank, KT3). Secondary antibodies were as follows: Alexa Fluor 488 goat anti-rabbit IgG (1:200; Invitrogen, A-11034); DyLight 650 goat anti-mouse IgA (1:200; Abcam, ab97014).

### Single-molecule FISH

Embryos and germlines were prepared through the methanol fixation step as described for immunofluorescence above. Slides were rehydrated by washing five times (all washes were performed in Coplin jars and carried out by briefly transferring slides from jar to jar) in PBS-Tween (PBS, 0.1% Tween 20), then fixed in PBS with 4% paraformaldehyde (Electron Microscopy Science, 15714) for 1 h in a humid chamber at room temperature. Slides were then washed four times in PBS-Tween, followed by twice in 2× SCC Buffer (Invitrogen, AM9763), then once in Wash Buffer (2× SCC, 10% formamide), then blocked in Hybridization Buffer (2× SCC, 10% formamide, 200 mg/ml bovine serum albumin, 2 mM ribonucleoside vanadyl complex, 0.2 mg/ml yeast total RNA, 10% dextran sulfate) in a humid chamber for at least 30 min at 37°C. Slides were then incubated with 50 nM probe solutions in Hybridization Buffer overnight in a humid chamber at 37°C. Slides were washed twice (30 min each) with Wash Buffer in a humid chamber at 37°C. Finally, slides were briefly washed twice in 2× SCC Buffer, once in PBS-Tween, and then twice in PBS before being mounted using Prolong Glass Antifade Mountant with NucBlue.

All probes were designed using the Stellaris Probe Designer from Biosearch Technologies, with the fluorophores Quasar570 or Quasar670. For histone transcript *in situ* hybridization, we designed probes based on the sequence of the *his-11* (H2B), *his-12* (H2A), *his-13* (H3) and *his-14* (H4) genes. These genes are in a cluster on chromosome II, but each probe is predicted to also recognize transcripts originating from other genes in the same histone class due to their high sequence similarity.

### Electron microscopy

Electron microscopy was carried out at the Johns Hopkins School of Medicine Microscope Facility. Dissected germlines from day 1 adults were processed for electron microscopy as previously described (Pitt et al., 2000). Thin sections, 60-90 nm, were cut with a diamond knife on a Leica UCT ultramicrotome and picked up with 2×1 mm Formvar copper slot grids. Grids were stained with 3% uranyl acetate and observed on a Thermo Fisher Talos L120C at 120 kV. Images were captured with a Thermo Fisher Ceta CCD camera (16 megapixel CMOS, 16-bit).

### RNAseq and small RNAseq

Staged adult *C. elegans* (72 h after L1 synchronization at 20°C) were collected, snap-frozen, and total RNA was extracted using TRIzol (Thermo Fisher Scientific, 15596026) and chloroform, using standard protocols.

Total RNA was hybridized to anti-sense oligonucleotides against *C. elegans* ribosomal RNA. Annealed anti-sense oligonucleotide-rRNA hybrids were digested using RNase H, as previously described (Duan et al., 2020). RNA sequencing libraries were prepared from 10 ng of rRNA-depleted RNA, from two independent biological replicates for each genotype, using the Illumina Stranded Total RNA kit (20040529) following the manufacturer's instructions and sequenced on a NovaSeq6000 system at the Johns Hopkins University School of Medicine Genetic Resources Core Facility. Read quality of all libraries was assessed using fastQC (v.0.11.9). Raw sequencing reads were aligned to the *C. elegans* reference genome ce 11 (WormBase version WS289) using STAR (Dobin et al., 2013). Total counts for each genomic feature were used for differential expression analysis using DESeq2 (Love et al., 2014). Statistically significant genes were chosen based on a Benjamini–Hochberg adjusted *P*-value of 0.05. Significantly misregulated transcripts were used to perform GO analysis, using the WormBase Enrichment Analysis Tool.

For sRNA sequencing libraries, 5 µg of total RNA was treated with 5′ polyphosphatase and 1 µg of purified, treated RNA was inputted into the Illumina TruSeq small RNA library prep kit (RS-200-0012) according to the manufacturer's instructions. Libraries were size selected, purified and sequenced on a NovaSeq6000 system at the Johns Hopkins University School of Medicine Genetic Resources Core Facility. Three independent biological replicates were sequenced for each genotype. Reads shorter than 18 nt and longer than 30 nt were discarded, and 5′ Illumina adaptor sequences were removed using the default settings of Cutadapt (Martin, 2011). Libraries were aligned to the reference genome ce11 (WormBase version WS289) using STAR (Dobin et al., 2013). Reads mapping to genomic features were counted using modified scripts based on previously described methods (Seroussi et al., 2023) and differential expression analysis was performed using DESeq2.

Upregulated and downregulated mRNAs for *nup214*Δ, *mip-1*Δ and *nup214*Δ *mip-1*Δ double mutants are listed in Tables S2-S7. Upregulated and downregulated sRNAs for *nup214*Δ *mip-1*Δ double mutants are listed in Tables S8 and S9.

### Fertility, brood size, and embryonic viability analysis

To measure fertility of *nup214*Δ, *mip-1*Δ and *glh-1*Δ*FGG* mutants (Fig. 6A), day 1 adults for each genotype were transferred to nine NNGM plates (eight worms per plate) and allowed to lay progeny for 2 h at 20°C. Adults were then removed, and three plates were incubated at 20°C, three at 25°C and three at 26°C. After 3 or 4 days, when all progeny reached adulthood, the progeny for each plate were scored by visual inspection using a stereoscope (Zeiss Stemi, 2000) as fertile (having a germline) or white sterile (lacking a germline). Sterility was measured as the number of sterile adults divided by the total number of progeny counted for each plate. All fertility measurements were repeated three times.

To measure the brood size of *nup214*Δ, *mip-1*Δ and *glh-1*Δ*FGG* mutants (Fig. 6B), six L4 larvae were transferred to individual NNGM plates and incubated at 20°C. On subsequent days, each worm was transferred to a new individual plate until it stopped laying progeny; any worm that crawled off the plate during this time was removed from analysis. All progeny were allowed to grow to adulthood at 20°C before being counted. All brood size experiments were repeated twice.

To measure embryonic viability of *nup214*Δ, *mip-1*Δ and *nup214*Δ *mip-1*Δ double mutants at 20°C versus 25°C (Fig. 6C), 20 day 1 adults for each genotype were transferred to two NNGM plates and allowed to lay progeny for 2 h at 20°C. Adults were then removed, and one plate was incubated at 20°C, while the other was transferred to 25°C. After 3 days, 30 fertile adults for each temperature were transferred to three NNGM plates (ten worms per plate) and allowed to lay progeny for 2 h at 20°C or 25°C. Adults were then removed and the number of embryos on each plate was counted. Plates were then incubated at 20°C or 25°C for 3 days and the number of adults per plate was counted. Embryonic viability was measured as the number of surviving adults or hatched larvae divided by the original number of embryos counted for each plate.

To measure lethality following *pos-1* RNAi (Fig. 7E), day 1 adults (4 and 8 h time points) or L4 larvae (24 h time point) were fed *pos-1* RNAi at 20°C. At the indicated time point, ten worms were transferred to three NNGM plates with OP50 (30 worms total) and allowed to lay progeny for 1 h at 20°C. Adults were then removed and the number of embryos per plate was counted. After 3 days at 20°C, the number of surviving adults and larvae was counted, and lethality was measured as described above.

### Imaging

For live imaging of germlines and somatic tissues, five staged adults were transferred to the middle well of a 3-chambered slide (Fisher Scientific, 30-2066A) in 10 μl of L-15 media with 10 mM levamisole. Then, 20 μm polystyrene beads (Bangs Laboratories Inc., PS07003) were added to support a coverslip (Marienfeld cat # 0107052). Animals were imaged using an inverted Zeiss Axio Observer with CSU-W1 SoRa spinning disk scan head (Yokogawa), 1×/2.8×/4× relay lens (Yokogawa) and an iXon Life 888 EMCCD camera (Andor) controlled by Slidebook 6.0 software (Intelligent Imaging Innovations). For low-resolution images of germlines, a 20 μm *z*-stack (1 μm step size) was captured using a 63× objective (Zeiss) with the 1× relay lens. For high-resolution images of germlines and somatic cells, 8 μm *z*-stacks (0.5 μm step size) were acquired using the 63× objective with the 4× relay lens. As *C. elegans* germlines are highly sensitive to imaging-induced stress (Elaswad et al., 2022), care was taken to avoid compression, and all animals were imaged only once and maintained on the slide for less than 10 min.

For live imaging of embryos, five young adults were transferred to 10 μl of L-15 media on a coverslip and dissected to release embryos. Then, 20 μm polystyrene beads were added to prevent compression, and the coverslip was inverted onto a microscope slide (Fisher Scientific, 12-550-403). Embryos were imaged as 15 μm *z*-stacks (1 μm step size), captured using the 63× objective with the 2.8× relay lens.

For imaging of fixed samples, microscopy was performed using a Zeiss Axio Imager Z1 with a CSU-W1 SoRA spinning disk scan head, 1×/2.8×/4× relay lens and an ORCA-Fusion BT digital CMOS camera (Hamamatsu). For low-resolution images of germlines, a 10 μm *z*-stack (0.5 μm step size) was captured using a 63× objective with the 1× relay lens. For higher-resolution images, 8 μm *z*-stacks (0.5 μm step size) were acquired using the 63× objective with the 4× relay lens.

Images were exported from SlideBook software and further analyzed using ImageJ or Imaris image analysis software. For presentation in figures, images were processed using ImageJ (Fiji) v.2.9.0 software, adjusting only the minimum/maximum brightness levels for clarity with identical leveling between all images in a figure panel. Images presented in figures are maximum intensity projections or single focal planes as indicated in the legends.

### Image analysis

All image quantification was performed using ImageJ (Fiji) v.2.9.0 software, with the exception of Fig. S4H, which was quantified using Imaris v.9.5.1 software.

To compare the Nup fluorescence of germline versus hypodermal (somatic) nuclei (Fig. 1C), the integrated density of two germline nuclei and one hypodermal nucleus within the same imaging plane were measured. Each measurement was background subtracted using fluorescence from regions just outside the nuclei. The background subtracted values for one germline nucleus and the hypodermal nucleus were then normalized to the value of the second germline nucleus.

To quantify the extent of nuclear pore clustering (Figs 1C, 2B, 4A,D), *z*-stacks of germlines were projected as 10 μm maximum intensity projections. Individual nuclei were cropped, avoiding clusters on the edge of nuclei, and the standard deviation of gray values for each nucleus was measured. In all experiments, experimental conditions were normalized to control conditions so that average value of the control condition=1.0.

To measure the area of nuclear pore clusters (Figs 1B, 3B and 4B), *z*-stacks of germlines were projected as 10 μm maximum intensity projections. Individual nuclei were cropped, avoiding clusters on the edge of nuclei. Images were smoothed once, then thresholded using the Otsu method to isolate individual clusters. Histograms of nuclear pore cluster areas were generated by summing the total area binned by the area of each cluster.

To quantify the area of nuclear pores overlapped by P granules (Figs 2A, 2B, 3B and 4D), single imaging planes were cropped to isolate individual nuclei. The nuclear pore and P granule channels were thresholded to isolate pores and pore clusters as well as P granules. The thresholded P granule image was then used to create a selection, which was then applied to the thresholded nuclear pore image to measure the area of nuclear pores overlapped by P granules. A similar protocol was used to measure Z granule area overlapped by P granules (Fig. S5G), with the thresholded P granule image used to create a selection that was applied to the thresholded Z granule image.

To quantify the distance between individual pores from electron micrographs (Fig. 4C), the line tool was used to measure the distance from the center of each pore to the center of the directly adjacent pores. Individual pores were approximated from fusion cites between the outer and inner nuclear envelope membrane, with a characteristic ∼100-120 nm diameter between fusion sites.

To quantify the distribution of Nup85 in condensates (Fig. S4H), *z*-stacks were exported to Imaris software. The ‘Surface’ tool was first used to isolate the -3 and -4 position oocytes from each germline, then the ‘Spot’ tool was used to isolate cytoplasmic foci. The percentage of protein present in condensates was measured as the intensity sum for all foci divided by the total intensity sum of the oocytes. To control for autofluorescent background, staged animals lacking fluorescent tags were imaged using identical imaging settings. The average intensity sum per volume was calculated for the -3 and -4 position oocytes of germlines lacking fluorescent tags and subtracted from the intensity sum measured for oocytes with tagged proteins.

To quantify the percentage of granules in the rachis (Fig. S5A,C), single imaging slices at the center of the germline were manually traced to isolate nuclei (edges of germline) from the rachis (center of germline). Each region was thresholded and the area of granules was measured. The summed area of granules in the rachis was then divided by the total granule area of the germline.

To compare the number of Mutator foci per nucleus (Fig. S5B), the number of Mutator foci were counted manually from a single imaging plane at the center of the nucleus. This central imaging plane was considered a proxy for the entire nucleus.

To quantify the center-of-mass distance between PGL-1 and ZNFX-1 granules (Fig. S5G), confocal stacks were cropped to isolate individual granules. The 3D objects counter plugin was then used to measure *x*, *y* and *z* coordinates for the center-of-mass for PGL-1 and ZNFX-1 granules. These coordinates were then used to calculate the center-of-mass distance for PGL-1 and ZNFX-1 granules.

To quantify cytoplasmic levels of histone mRNA (Fig. 7B,C), the integrated density for the histone RNA as well as *puf-5* (a control mRNA levels of which do not change across mutants, see Tables S2-S7), was measured in the rachis of the pachytene germline just after the transition zone. For both histone and *puf-5* signals, a background measurement was subtracted using the average fluorescence of three regions just outside of the germline. The background subtracted histone signal was then normalized to that of *puf-5*, and the resulting values were normalized so that the average signal such that wild type=1.0.

### Statistical analysis

All statistical tests were performed using GraphPad Prism v.10.2.3 software. For comparison of two groups, significance was determined using an unpaired *t*-test. For comparison of three or more groups, significance was determined using a one-way ANOVA. In all figures, error bars represent 95% confidence intervals (CI). ‘ns’ indicates not significant; *P<0.05; **P<0.01; ***P<0.001; ****P<0.0001.

## Supplementary Material



10.1242/develop.204585_sup1 Supplementary information

Table S1. *C. elegans* strains generated and used in this study.

Table S2. List of upregulated genes in *nup214*Δ relative to N2

Table S3. List of downregulated genes in *nup214*Δ relative to N2

Table S4. List of upregulated genes in *mip-1*Δ relative to N2

Table S5. List of downregulated genes in *mip-1*Δ relative to N2

Table S6. List of upregulated genes in *nup214*Δ *mip-1*Δ relative to N2

Table S7. List of downregulated genes in *nup214*Δ *mip-1*Δ relative to N2

Table S8. List of upregulated sRNAs in *nup214*Δ *mip-1*Δ relative to N2

Table S9. List of downregulated sRNAs in *nup214*Δ *mip-1*Δ relative to N2
