# Supplementary material for: FG repeats drive co-clustering of nuclear pores and P granules in the C. elegans germline
Source: Development. 2025 Mar 27;152(6):dev204585. doi: 10.1242/dev.204585 (PMC12050070; doi:10.1242/dev.204585)
Supplement: Supplementary information [file develop-152-204585-s1.pdf]

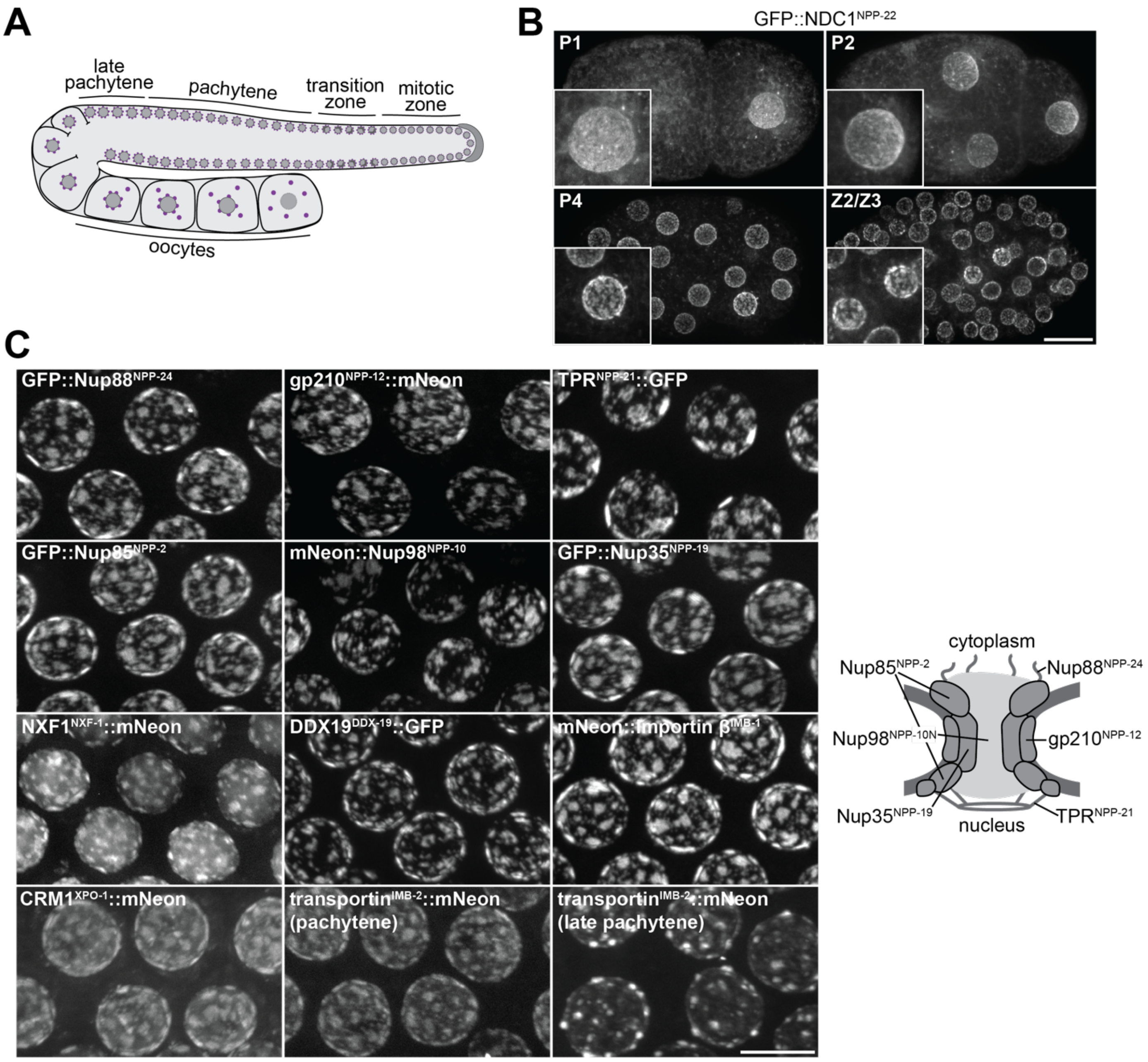

Fig. S1. Nucleoporins representing all pore subcomplexes are clustered in the pachytene germline

A. Schematic depicting the regions of the *C. elegans* distal germline. Germ cell nuclei proliferate in the mitotic zone, enter meiotic prophase in the transition zone, and progress to pachytene while in a syncytium around a shared central cytoplasm (the rachis). At the bend, nuclei bud from the syncytium to form individualized oocytes that stack in a single row in the proximal germline. P granules are denoted in purple.

B. Representative confocal micrographs of GFP::NDC1^{NPP-22} in *C. elegans* embryos. Germ (P) cell nuclei are magnified in boxes at the bottom left.

C. Left: Representative confocal micrographs of the indicated CRISPR-tagged Nup or nuclear transport receptor (NTR) in the pachytene germline. Notably, transportin^{IMB-2} is enriched in perinuclear foci specifically in the late pachytene germline. Right: Schematic of the nuclear pore complex showing the localization of each tagged Nup.

All images are maximum intensity projections. Scale bars = 10 μm (panel B) or 5 μm (panel C).

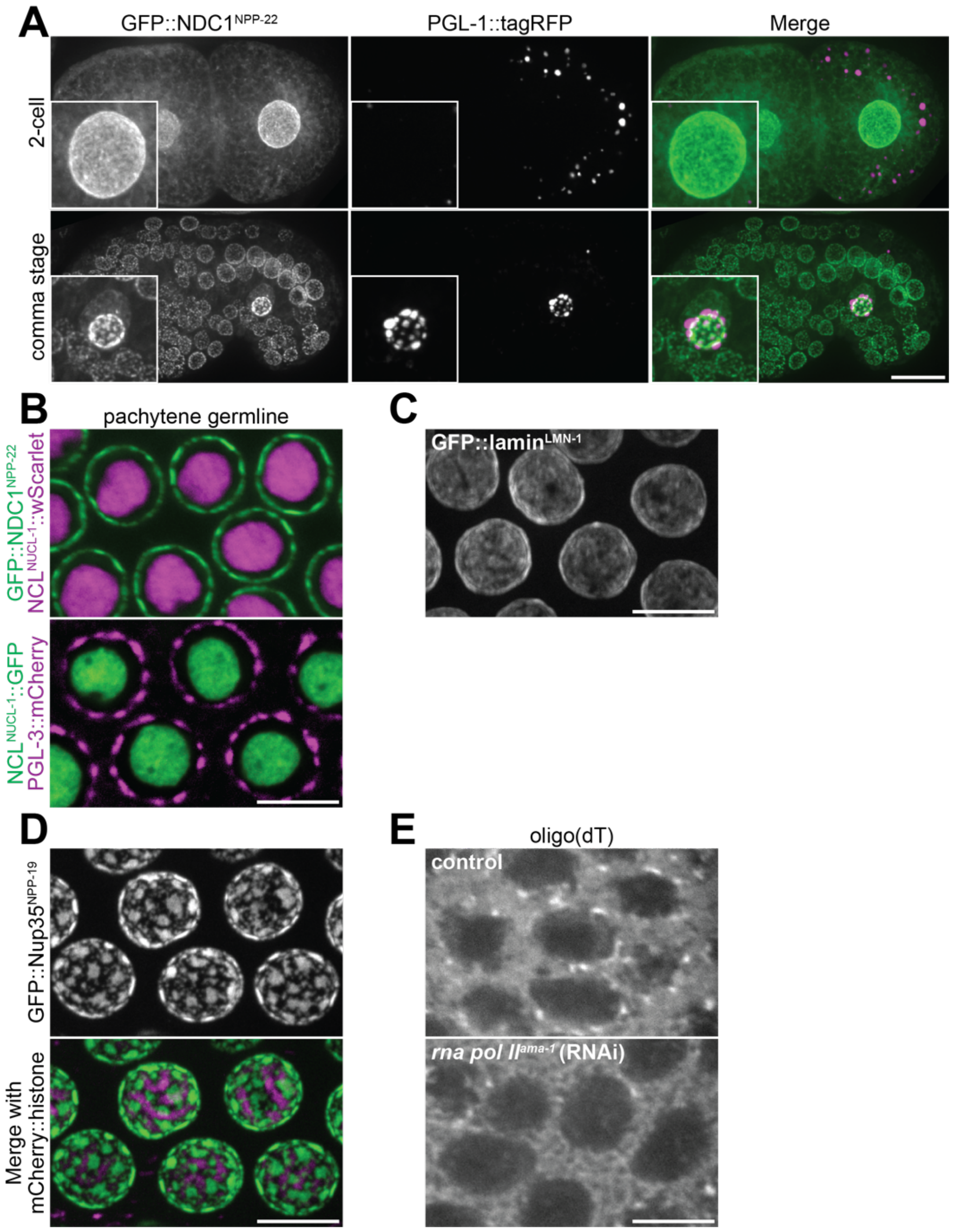

Fig. S2. Distribution of nuclear pore clusters relative to P granules, nucleoli, and chromatin

A. Representative confocal micrographs GFP::NDC1$^{NPP\text{-}22}$ and the P granule marker PGL-1::tagRFP in an early (2-cell) embryo versus an older (comma stage) embryo. Germ (P) cell nuclei are magnified in boxes at the bottom left.

B. Representative confocal micrographs of pachytene nuclei co-expressing GFP::NDC1$^{NPP\text{-}22}$, the nucleolar marker NCL$^{NUCL\text{-}1}$, and the P granule marker PGL-3::mCherry as indicated.
C. Representative confocal micrograph of nuclei expressing GFP::lamin$^{LMN\text{-}1}$ in the pachytene germline.
D. Representative confocal micrographs of nuclei expressing GFP::Nup35$^{NPP\text{-}19}$ and a mCherry::histone transgene to mark chromatin.
E. Representative confocal micrographs showing the distribution of polyadenylated mRNA (oligo(dT)) in pachytene comparing control and germlines depleted of the RNA polymerase II subunit AMA-1 by RNAi.

All images are maximum intensity projections, with the exception of panels B and E which are single focal planes. Scale bars = 10 μm (panel A) or 5 μm (all other panels).

Development • Supplementary information

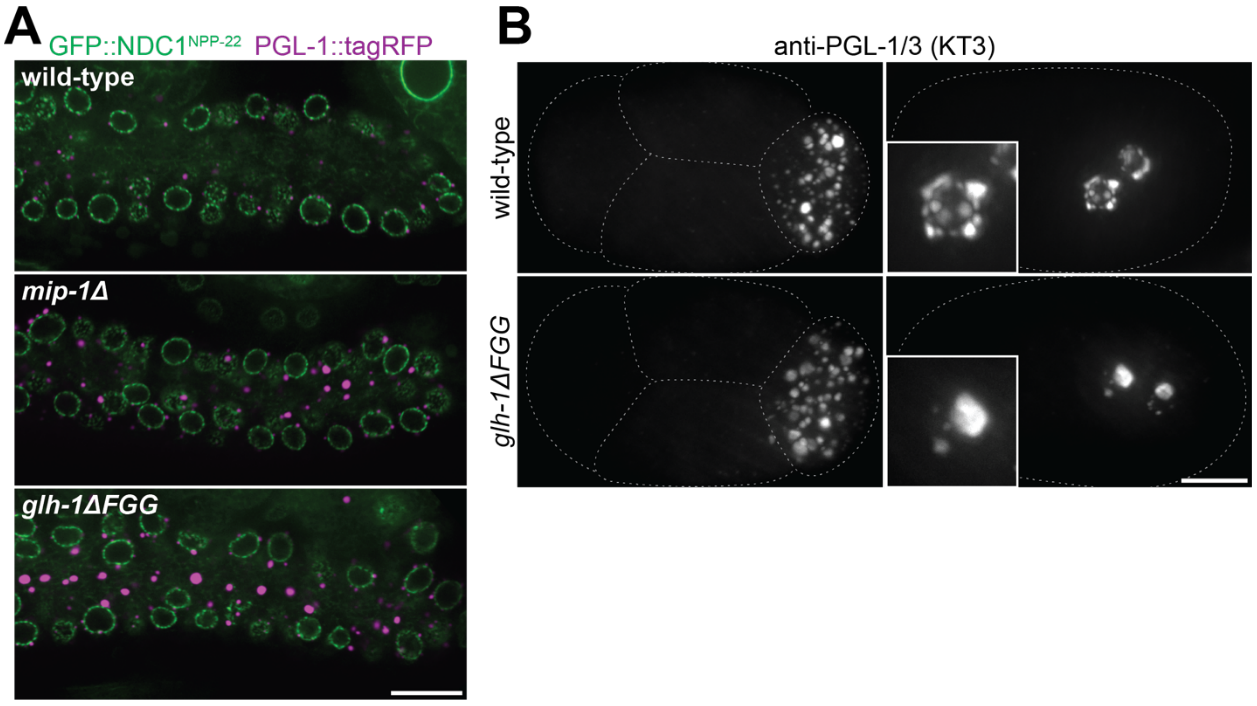

Fig. S3. P granule association with the nuclear envelope is disrupted in *mip-1*Δ and *glh-1*Δ*FGG* mutants

A. Representative confocal micrographs of nuclei in the mitotic region of the germline expressing GFP::NDC1$^{NPP\text{-}22}$ and the P granule marker PGL-1::tagRFP in the indicated genotypes.
B. Representative confocal micrographs of 4-cell (left) and ~100-cell (right) embryos immunostained with the P granule antibody KT3 (recognizing PGL-1 and PGL-3 proteins). One of the two germ cells of each ~100-cell embryo is magnified in the insets. The distribution of P granules around nuclei is disrupted at the 100-cell stage in *glh-1*Δ*FGG* mutants.

Images in panel A are single focal planes, and images in panel B are maximum intensity projections. Scale bars = 10 µm.

Development • Supplementary information

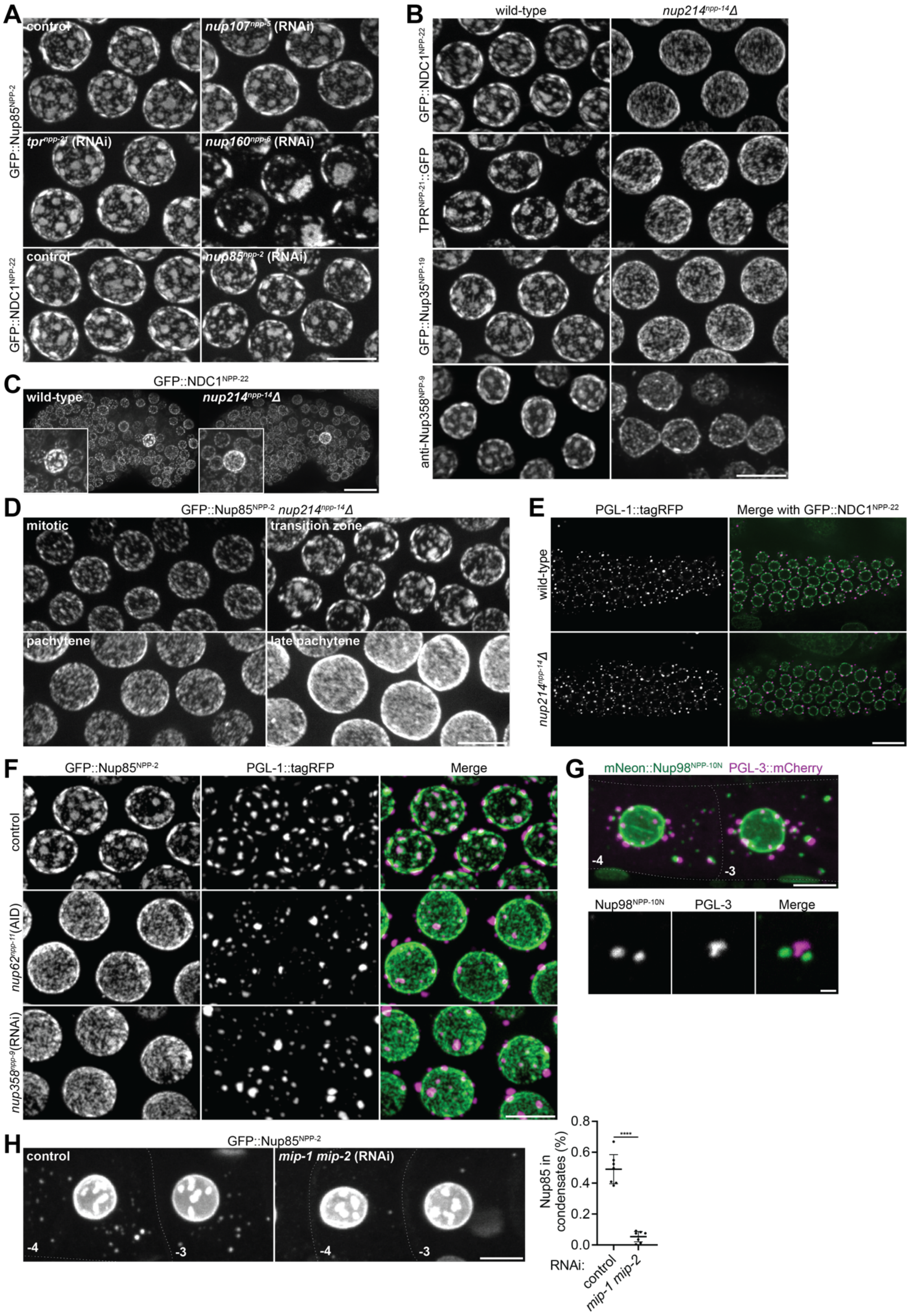
A
control
nup107npp-5 (RNAi)
GFP::Nup85NPP-2
tprnpp-21 (RNAi)
nup160npp-6 (RNAi)
control
nup85npp-2 (RNAi)
GFP::NDC1NPP-22
B
wild-type
nup214npp-14Δ
GFP::NDC1NPP-22
TPRNPP-21::GFP
GFP::Nup35NPP-19
anti-Nup358NPP-9
C
GFP::NDC1NPP-22
wild-type
nup214npp-14Δ
D
GFP::Nup85NPP-2 nup214npp-14Δ
mitotic
transition zone
pachytene
late pachytene
E
PGL-1::tagRFP
Merge with GFP::NDC1NPP-22
wild-type
nup214npp-14Δ
F
GFP::Nup85NPP-2
PGL-1::tagRFP
Merge
control
nup62npp-11(AID)
nup358npp-9(RNAi)
G
mNeon::Nup98NPP-10N
PGL-3::mCherry
-4
-3
Nup98NPP-10N
PGL-3
Merge
H
GFP::Nup85NPP-2
control
mip-1 mip-2 (RNAi)
-4
-3
-4
-3
0.8
0.6
0.4
0.2
0.0
****
Nup85 in condensates (%)
RNAi:
control
mip-1 mip-2

Fig. S4. P granules contribute to Nup condensation at the nuclear membrane and in the cytoplasm of oocytes

A. Representative confocal micrographs of pachytene nuclei expressing GFP::Nup85$^{NPP\text{-}2}$ or GFP::NDC1$^{NPP\text{-}22}$ following RNAi-mediated depletion of the indicated Nups.
B. Representative confocal micrographs of pachytene nuclei expressing GFP::NDC1$^{NPP\text{-}22}$, TPR$^{NPP\text{-}21}$::GFP, GFP::Nup35$^{NPP\text{-}19}$, or stained for endogenous Nup358$^{NPP\text{-}9}$ in wild-type versus *nup214*$^{npp\text{-}14}\Delta$ mutant germlines.
C. Representative confocal micrographs of GFP::NDC1$^{NPP\text{-}22}$ in wild-type versus *nup214*$^{npp\text{-}14}\Delta$ comma stage embryos. P cell nuclei are magnified in boxes at the bottom left.
D. Representative confocal micrographs of nuclei expressing GFP::Nup85$^{NPP\text{-}2}$ in four regions of *nup214*$^{npp\text{-}14}\Delta$ mutant germlines: the mitotic zone, transition zone, mid-pachytene, and late pachytene.
E. Representative confocal micrographs of nuclei expressing the P granule marker PGL-1::tagRFP and GFP::NDC1$^{NPP\text{-}22}$ in the mitotic germline of wild-type versus *nup214*$^{npp\text{-}14}\Delta$ mutants.
F. Representative confocal micrographs of pachytene nuclei expressing GFP::Nup85$^{NPP\text{-}2}$ and PGL-1::tagRFP in control germlines versus those depleted of Nup62$^{NPP\text{-}11}$ or Nup358$^{NPP\text{-}9}$.
G. Top: Representative confocal micrographs of wild-type -3 and -4 position oocytes expressing mNeonGreen::Nup98$^{NPP\text{-}10N}$ and the P granule marker PGL-3::mCherry. Bottom: High resolution confocal micrographs of a single cytoplasmic P granule with two distinct Nup condensates.
H. Left: Representative confocal micrographs of -3 and -4 position oocytes expressing GFP::Nup85$^{NPP\text{-}2}$ in control germlines or following RNAi depletion of MIP-1 and MIP-2. Right: Quantification of the percent of Nup85$^{NPP\text{-}2}$ in condensates under each indicated condition. Error bars represent 95% CI for n = 7 germlines.

All images are maximum intensity projections, with the exception of panels E and G, which are single focal planes. Scale bars = 10 µm (panels C, E, G (top), and H), 1 µm (panel G (bottom)), or 5 µm (all other panels). ****P<0.0001 (significance was determined using an unpaired t-test).

Development • Supplementary information

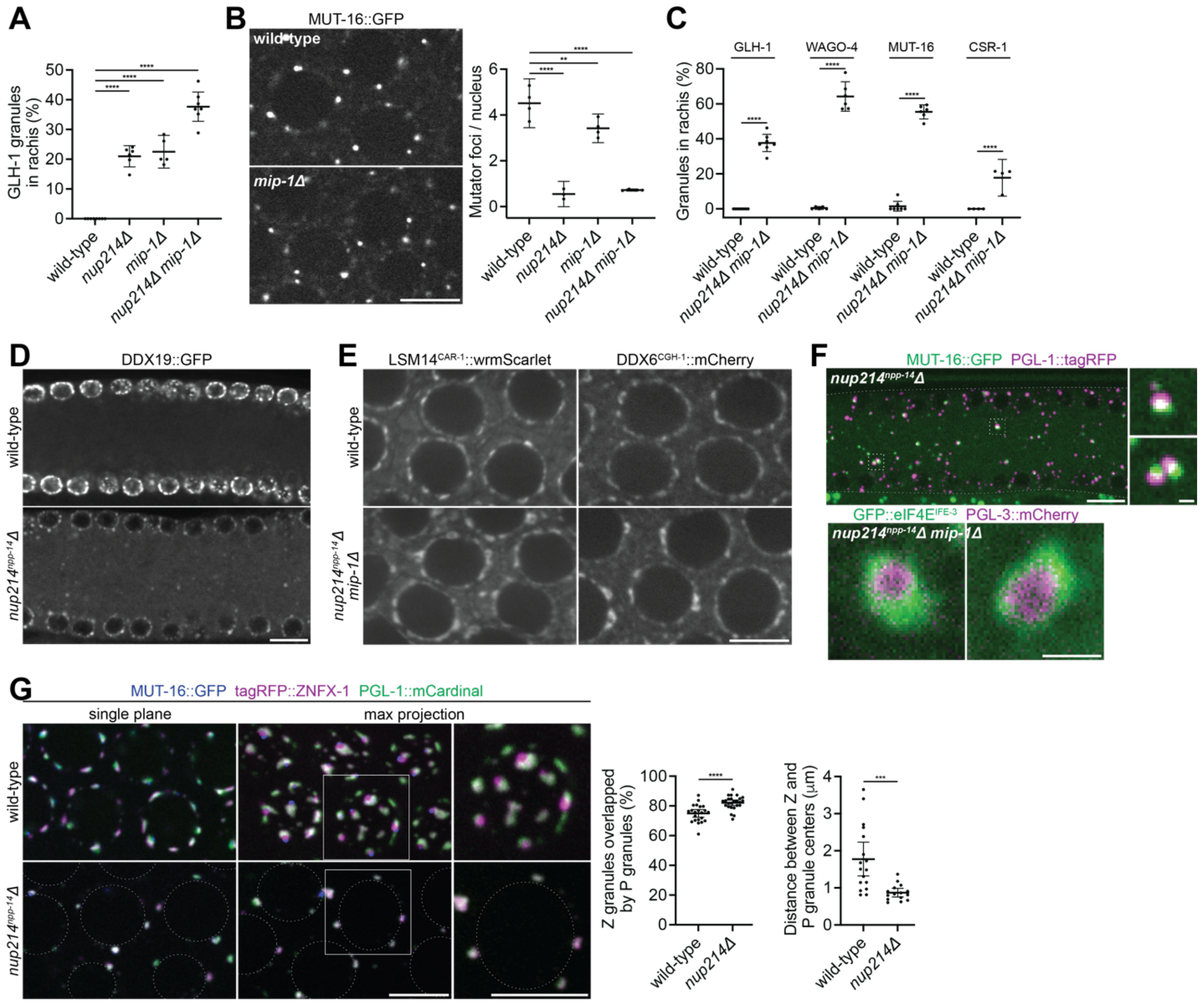

Fig. S5. Localization of nuage condensates in *nup214^{npp-14}*Δ and *mip-1*Δ mutants

A. Quantification of the percent of GLH-1::GFP granules in the cytoplasm (rachis) in wild-type versus *nup214^{npp-14}*Δ and *mip-1*Δ mutant germlines. Error bars represent 95% CI for *n* ≥ 5 germlines.
B. Left: Representative confocal micrographs of pachytene nuclei expressing the Mutator foci marker MUT-16::GFP in wild-type versus *mip-1*Δ mutants. Right: Quantification of the number of perinuclear MUT-16 foci in the middle plane of the nucleus. Error bars represent 95% CI for *n* ≥ 13 nuclei from 3 germlines.
C. Quantification of the percent of P granules (GLH-1), Z granules (WAGO-4), Mutator foci (MUT-16) or CSR-1 granules in the cytoplasm (rachis) in wild-type

Development • Supplementary information

versus *nup214*$^{npp-14}$Δ *mip-1*Δ mutant germlines. Error bars represent 95% CI for *n* ≥ 4 germlines. Quantification for GLH-1 is reproduced from Figure S5A.

D. Representative confocal micrographs of pachytene germlines expressing the D granule marker DDX19::GFP in wild-type versus *nup214*Δ mutants.
E. Representative confocal micrographs of pachytene nuclei expressing the P-body markers LSM14^{CAR-1}::wrmScarlet and DDX6^{CGH-1}::mCherry in wild-type versus *nup214*$^{npp-14}$Δ *mip-1*Δ mutant germlines.
F. Top: Representative confocal micrographs of the cross-sectioned pachytene region expressing MUT-16::GFP and the P granule marker PGL-1::tagRFP in a *nup214*$^{npp-14}$Δ mutant. The regions indicated by white boxes are magnified to the right. Bottom: Representative confocal micrographs of cytoplasmic P granules encircled by P-bodies in *nup214*$^{npp-14}$Δ *mip-1*Δ mutant germlines. P granules and P-bodies are marked by PGL-3::mCherry and GFP::eIF4E^{IFE-3}, respectively.
G. Left: Representative confocal micrographs of pachytene nuclei expressing MUT-16::GFP, the Z granule marker tagRFP::ZNXF-1, or PGL-1::mCardinal in wild-type versus *nup214*$^{npp-14}$Δ mutant germlines. Middle: Quantification of the total area occupied by ZNFX-1 that is overlapped by PGL-1. Error bars represent 95% CI for *n* ≥ 22 granules. Right: Quantification of the distance between centers of mass for P granules (PGL-1) and Z granules (ZNFX-1) in wild-type versus *nup214*$^{npp-14}$Δ mutants. Error bars represent 95% CI for *n* ≥ 15 granules.

All images are single focal planes, with the exception of the noted images in panel G. Scale bars = 10 µm (panels D and F (top left)), 1 µm (panel F (top right and bottom), or 5 µm (all other panels). ****P<0.0001; ***P<0.001; **P<0.01 (significance was determined using a one-way ANOVA (panels A and B) or an unpaired t-test (panels C and G).

Development • Supplementary information

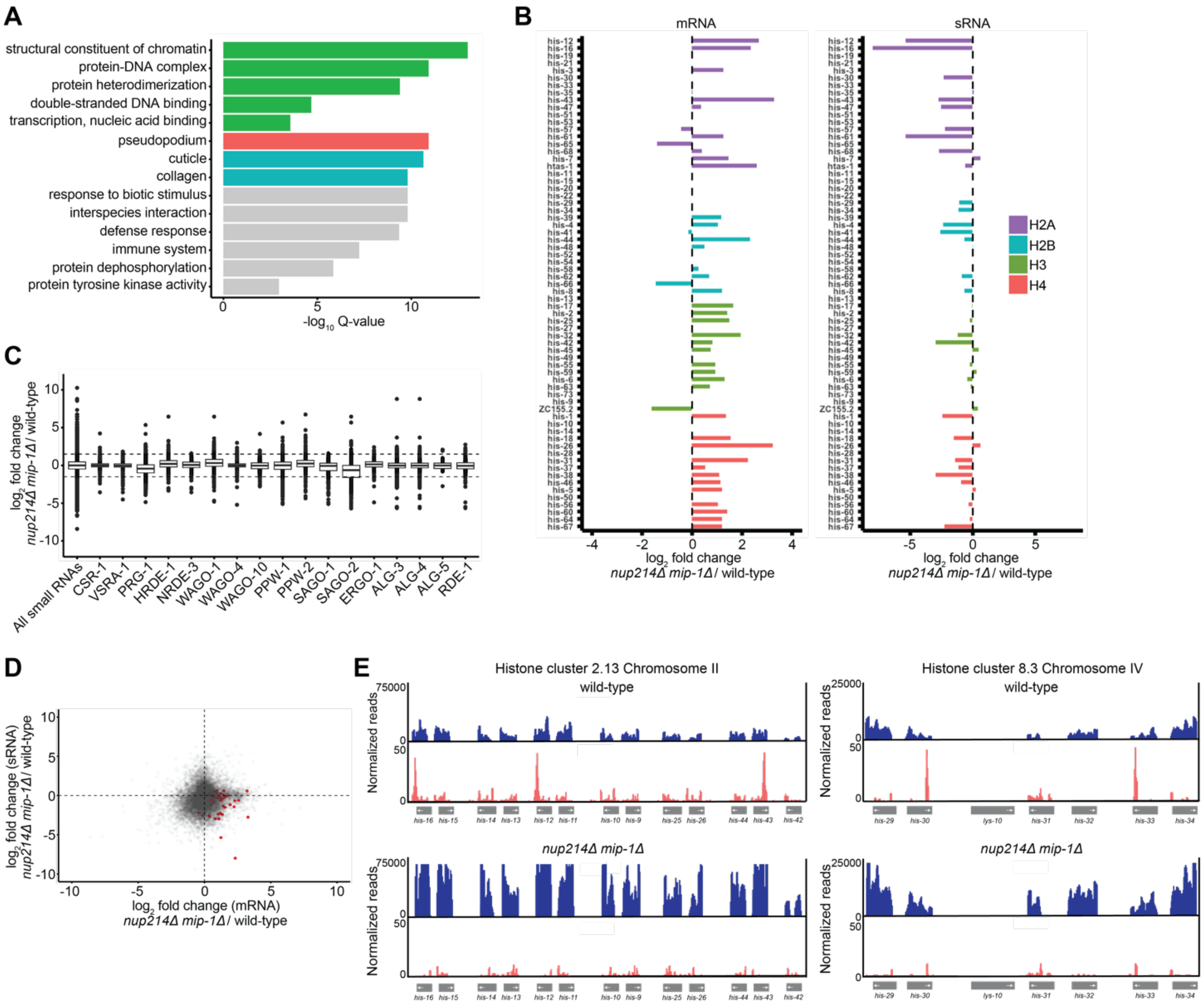

Fig. S6. Replication-dependent histone transcripts are upregulated, and anti-histone sRNAs are reduced, in *nup214^{npp-14}*Δ *mip-1*Δ mutants

A. Gene ontology (GO) analysis of transcripts upregulated in *nup214^{npp-14}*Δ *mip-1*Δ mutants relative to wild-type. Terms that include replication-dependent histones are denoted in green. Pseudopodium (sperm genes), cuticle, and collagen categories were also reported as upregulated in *mip-1*Δ mutants by Price et al., 2023.
B. Graph showing the log_2 fold change of mRNAs and sRNAs in *nup214^{npp-14}*Δ *mip-1*Δ mutants relative to wild-type for all replication-dependent histone genes.
C. Graph showing log_2 fold change of 31,347 detected sRNAs binned by their associated Argonaute (Seroussi et al. 2023). Dashed lines denote the log_2 fold changes used to define significant mis-regulation.
D. Graph showing the fold change in mRNA (x-axis) versus sRNA (y-axis) abundance comparing *nup214^{npp-14}*Δ *mip-1*Δ to wild-type (log_2 fold change (*nup214^{npp-14}*Δ *mip-1*Δ / wild-type)). Replication-dependent histone transcripts are highlighted in red.
E. Genome browser views of two histone gene clusters, showing mRNA (blue) and sRNA (red) levels in wild-type versus *nup214^{npp-14}*Δ *mip-1*Δ mutants.

Development • Supplementary information

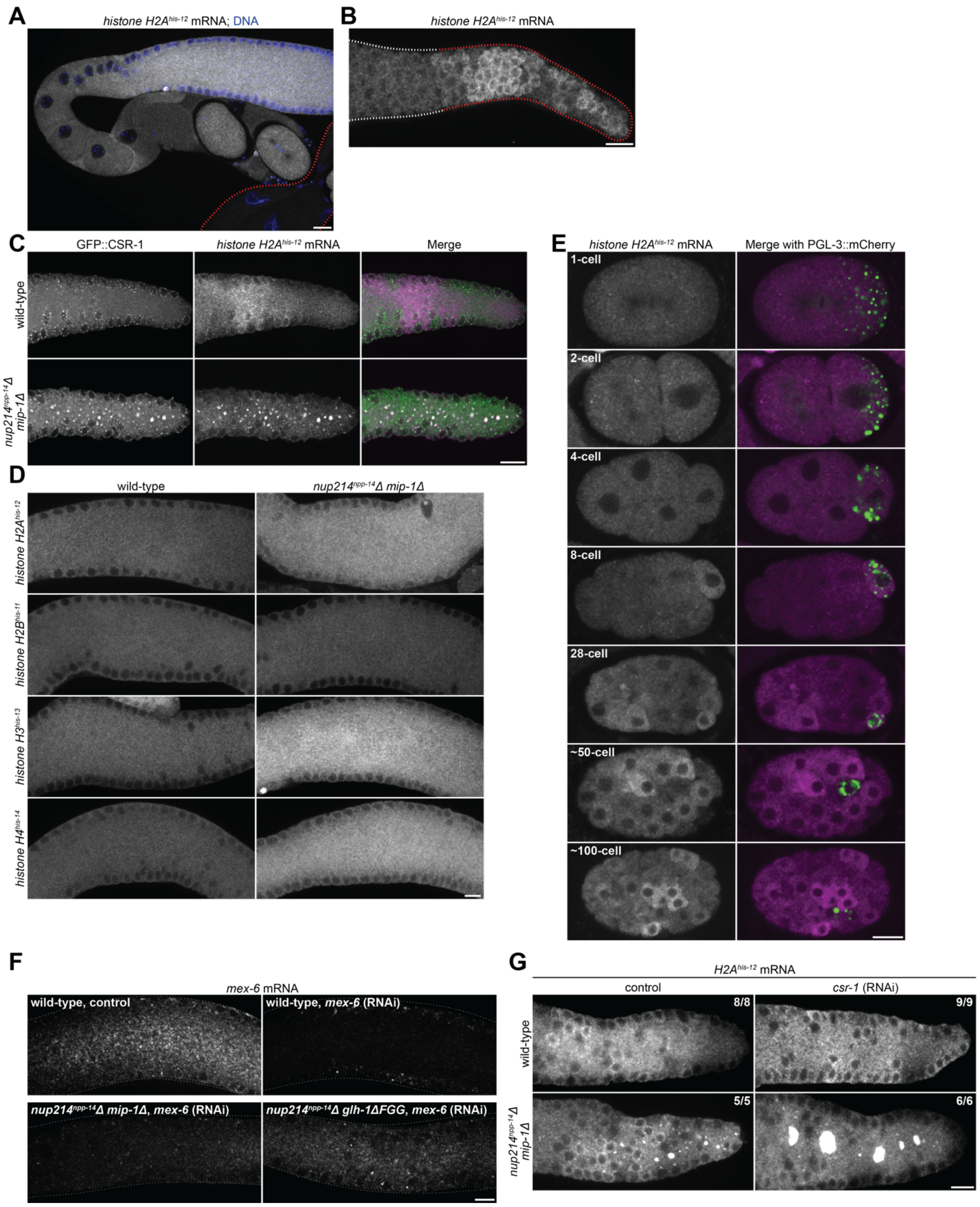
A
histone H2Ahis-12 mRNA; DNA
B
histone H2Ahis-12 mRNA
C
GFP::CSR-1
histone H2Ahis-12 mRNA
Merge
wild-type
nup214npp-14Δ mip-1Δ
D
wild-type
nup214npp-14Δ mip-1Δ
histone H2Ahis-12
histone H2Bhis-11
histone H3his-13
histone H4his-14
E
histone H2Ahis-12 mRNA
Merge with PGL-3::mCherry
1-cell
2-cell
4-cell
8-cell
28-cell
~50-cell
~100-cell
F
mex-6 mRNA
wild-type, control
wild-type, mex-6 (RNAi)
nup214npp-14Δ mip-1Δ, mex-6 (RNAi)
nup214npp-14Δ glh-1ΔFGG, mex-6 (RNAi)
G
H2Ahis-12 mRNA
control
csr-1 (RNAi)
wild-type
nup214npp-14Δ mip-1Δ
8/8
9/9
5/5
6/6

Fig. S7. Distribution of histone transcripts in wild-type and mutant germlines and embryos

A. Representative confocal micrograph of a wild-type dissected germline, uterus with embryos, and intestine (red dashed line) hybridized to probe against *histone* $H2A^{his\text{-}12}$ mRNA. DAPI-stained DNA is in blue. No *histone* signal is detected in the intestine.
B. Representative confocal micrograph of the distal tip of a wild-type germline hybridized to a probe against *histone* $H2A^{his\text{-}12}$ mRNA. Red and white dashed lines denote the mitotic zone and transition zone, respectively. Clusters of germ cells show elevated levels of histone transcripts consistent with S phase-specific transcription.
C. Representative confocal micrographs of the distal tips of wild-type and $nup214^{npp\text{-}14}\Delta$ *mip-1*Δ germlines hybridized to a probe against *histone* $H2A^{his\text{-}12}$ mRNA and expressing the Argonaute GFP::CSR-1 to identify P granules.
D. Representative confocal micrographs of the pachytene region of wild-type and $nup214^{npp\text{-}14}\Delta$ *mip-1*Δ germlines hybridized to probes against the indicated histones. Quantification of the different levels is shown in Fig. 7B. Note that no change was detected in this assay for histone H2B, which also showed the weakest change compared to other histones by RNAseq as shown in Fig. S6B.
E. Representative confocal micrographs of wild-type embryos of the indicated stage hybridized to a probe against *histone* $H2A^{his\text{-}12}$ mRNA and expressing the P granule marker PGL-3::mCherry. Uniform distribution of (presumably) maternal histone transcripts is observed until the 8-cell stage. At that stage, replication-dependent transcription/degradation initiates in synchrony with the cell cycle in all blastomeres.
F. Representative confocal micrographs of germlines (pachytene region) of indicated genotype hybridized to a probe against *mex-6* mRNA under control conditions or following 24 hr *mex-6* RNAi.
G. Representative confocal micrographs of the distal tip of germlines of the indicated genotype hybridized to a probe against $H2A^{his\text{-}12}$ mRNA. The fraction of germlines with the representative phenotype is denoted in the upper right of each image. *csr-1* RNAi leads to enlarged P granules as previously reported (Updike and Strome 2009) but is not sufficient to cause accumulation of $H2A^{his\text{-}12}$ mRNA in P granules on its own, nor does it suppress accumulation in $nup214^{npp\text{-}14}\Delta$ *mip-1*Δ mutants. We do not know whether the apparent enhancement by *csr-1* RNAi of histone mRNA accumulation in P granules in $nup214^{npp\text{-}14}\Delta$ *mip-1*Δ germlines is a visual artefact due to *csr-1*'s effect on P granules (which become fused and larger in *csr-1* mutants), or is a reflection of *csr-1*'s role on histone mRNA processing, or a combination.

All images are single focal planes. Scale bars = 10 µm

Development • Supplementary information

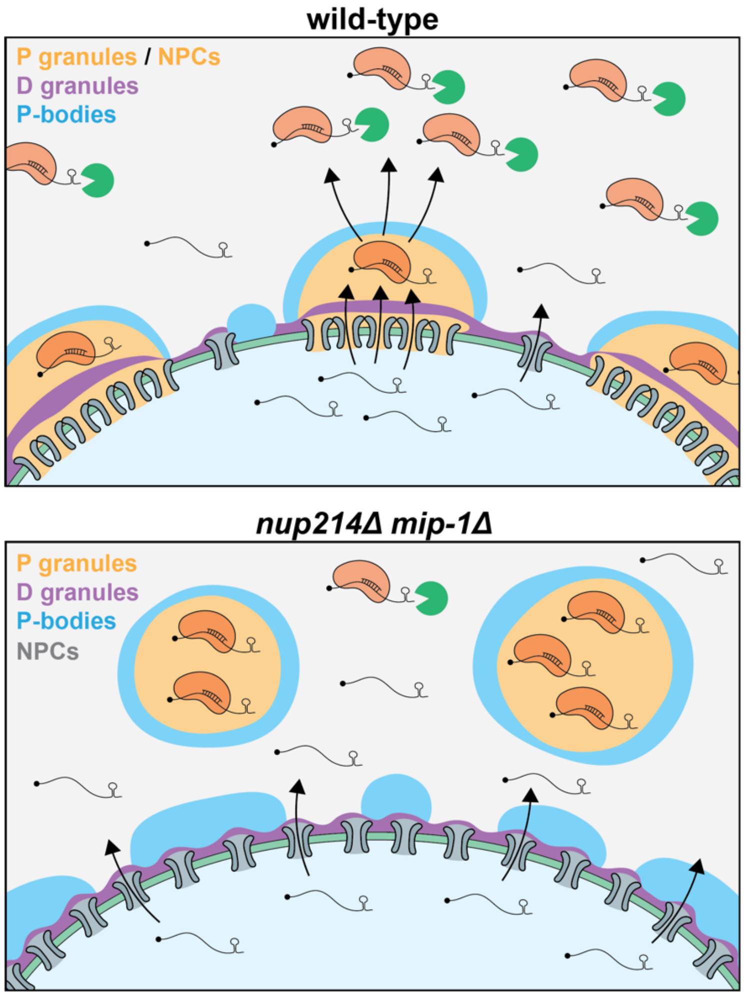

Fig. S8. Working model
Working model comparing the distribution of histone transcripts in a wild-type cycling germ cell compared to a *nup214*Δ *mip-1*Δ mutant germ cell where P granules are detached from nuclei.

Wild-type: Cohesive interactions among cytoplasmic FG-Nups bridge adjacent nuclear pores to form clusters. These clusters are favorable binding surfaces for P granules, which are directly connected to nuclear pores and experience rapid vectorial flow of nascent transcripts. Most histone transcripts traverse a P granule where they are recognized by Argonaute/sRNA complexes and tagged for sRNA amplification and degradation in the cytoplasm.

*nup214*Δ *mip-1*Δ mutants: Loss of Nup214 disrupts the FG-Nup interaction network causing nuclear pores to assume a uniform distribution across the surface of the nuclear envelope. P granules are not connected to nuclear pores and experience slower RNA flux. Some histone transcripts do not enter P granules and thus avoid being marked for sRNA amplification/degradation resulting in higher accumulation in the cytoplasm. Histone transcripts that enter the granules accumulate in the granule for a longer time, temporarily avoiding degradation in the cytoplasm.

Table S1. *C. elegans* strains generated and used in this study.

Available for download at
https://journals.biologists.com/dev/article-lookup/doi/10.1242/dev.204585#supplementary-data

Table S2. List of upregulated genes in *nup214*Δ relative to N2

Available for download at
https://journals.biologists.com/dev/article-lookup/doi/10.1242/dev.204585#supplementary-data

Table S3. List of downregulated genes in *nup214*Δ relative to N2

Available for download at
https://journals.biologists.com/dev/article-lookup/doi/10.1242/dev.204585#supplementary-data

Table S4. List of upregulated genes in *mip-1*Δ relative to N2

Available for download at
https://journals.biologists.com/dev/article-lookup/doi/10.1242/dev.204585#supplementary-data

Table S5. List of downregulated genes in *mip-1*Δ relative to N2

Available for download at
https://journals.biologists.com/dev/article-lookup/doi/10.1242/dev.204585#supplementary-data

Table S6. List of upregulated genes in *nup214*Δ *mip-1*Δ relative to N2

Available for download at
https://journals.biologists.com/dev/article-lookup/doi/10.1242/dev.204585#supplementary-data

Table S7. List of downregulated genes in *nup214*Δ *mip-1*Δ relative to N2

Available for download at
https://journals.biologists.com/dev/article-lookup/doi/10.1242/dev.204585#supplementary-data

Available for download at
https://journals.biologists.com/dev/article-lookup/doi/10.1242/dev.204585#supplementary-data

Table S8. List of upregulated sRNAs in *nup214*Δ *mip-1*Δ relative to N2

Available for download at
https://journals.biologists.com/dev/article-lookup/doi/10.1242/dev.204585#supplementary-data

Table S9. List of downregulated sRNAs in *nup214*Δ *mip-1*Δ relative to N2

Available for download at
https://journals.biologists.com/dev/article-lookup/doi/10.1242/dev.204585#supplementary-data
