# Supplementary material for: FG repeats drive co-clustering of nuclear pores and P granules in the C. elegans germline
Source: Development. 2025 Mar 27;152(6):dev204585. doi: 10.1242/dev.204585 (PMC12050070; doi:10.1242/dev.204585)
Supplement: Supplementary file 1 [file develop-152-204585_review_history.pdf]

FG repeats drive co-clustering of nuclear pores and P granules in the *C. elegans* germline

Laura L. Thomas, Devavrat Bodas, Geraldine Seydoux
DOI: 10.1242/dev.204585

Editor: Swathi Arur

Review timeline

Original submission:	13 December 2024
Editorial decision:	9 January 2025
First revision received:	7 February 2025
Accepted:	19 February 2025

Original submission

First decision letter

MS ID#: dev.204585

MS Title: FG repeats drive co-clustering of nuclear pores and P granules in the C. elegans germline

Authors: Laura L. Thomas; Devavrat Bodas; Geraldine Seydoux
Article Type: Research Article

I have now received all the referees reports on the above manuscript, and have reached a decision. The referees' comments are appended below, or you can access them online: please go to.

The overall evaluation is positive and we would like to publish a revised manuscript in Development. However, the reviewers recommend textual changes to the manuscript, including softening of some of the claims and similar suggestions which will further improve the rigor and clarity of the study. Please attend to all of the reviewers' comments in your revised manuscript and detail them in your point-by-point response. If you do not agree with any of their criticisms or suggestions explain clearly why this is so.

Reviewer 1

Advance summary and potential significance to field

This study by Thomas, Bodas, and Seydoux, titled "FG repeats drive co-clustering of nuclear pores and P granules in the C. elegans germline," substantiates several hypothesized roles of FG-repeat proteins in the context of nuclear pores and germ granules. It also presents novel findings addressing whether P granule functions depend on their localization at the nuclear periphery. The conclusions are supported by robust and well-controlled evidence. The number of precision-edited C. elegans lines generated is impressive, and the imaging quality is exceptional. The study's findings will significantly impact and interest researchers studying germ granules, the in vivo organization of biomolecular condensates, and small RNA regulation. The following critiques suggest minor revisions to help clarify and further strengthen the study's conclusions.

Comments for the author

Line 111 - Soften language - saying the activity is fully dispensable is a high bar.

© 2025. Published by The Company of Biologists under the terms of the Creative Commons Attribution License (https://creativecommons.org/licenses/by/4.0/).

Line 234 - The authors may want to speculate on why these clustering observations are so different between the mitotic and pachytene cells.
Line 241 - The term "partially independent" is hard to interpret and implies that the results could also be "partially dependent."
Line 248 - The initial reference to D granules might pose a challenge since the Huang paper is still on BioRxiv, and many readers may not recognize that D granules refer to DDX-19 granules located at the interface between NPCs and P granules. It may be helpful to provide a broader explanation here. This issue arises again in Figure 5A, where CSR-1 is labeled as a D-granule marker. In the Huang paper, multiple genes are associated with P granules and additional granules, with CSR-1 being just one example. However, at this stage, it seems premature to classify CSR-1 as a D-granule component. The observed retention of some CSR-1 at the nuclear envelope could be explained by its association with P and D granules, but labeling it definitively as a D-granule component does not seem entirely accurate.
Line 263 - Indicate this as eIF4EIFE-3 as IFE-1 and IFE-3 don't completely overlap.
Line 272 and S5E - This may require a center-of-mass analysis instead of colocalization, which could be performed using the same images, as P and Z granules show partial overlap in wild-type (WT) conditions. This overlap might be due to exposure or differences in the reporters. Additionally, the sizes of these granules are likely quite different when detached. A center-of-mass analysis would offer a clearer understanding of the extent to which P and Z granules overlap when detached. I would also be interested in hearing the authors speculate on the significance of this observation.
Line 295, 6, and S6A - Rochester et al. (2022) identified the same three classes of genes—sperm, stress (HSP-16), and replication-dependent histones—as being enriched in their analysis of GLH-1-dependent differential translation (polysome vs. total mRNA; see their Fig. 1B and Supplemental Table). While it is true that replication-dependent histones can be overlooked with polyA selection, sufficient sequencing depth can still detect enough of them for differential expression analysis.
Fig S2B - Specify that these are pachytene
Figure 4C - The nup214 deletion micrographs appear to show additional nuclear pores that are not marked with white arrows. The authors should clarify the criteria used to identify and label nuclear pores in these images.
Figure 6 heading. Consider removing glh-1FGG from the heading as it doesn't seem expression profiling was done on this mutant to show elevated levels of histone transcripts
Fig 7B - Quantification is of rachis images, but images are not shown. Because 7A shows mitotic images, it may be incorrectly assumed that the quant in B is for these mitotic images.
Fig 7E - change axis and legend - should not be viability %, but lethality ratio.

Reviewer 2

Advance summary and potential significance to field

In this manuscript by Thomas et al., the authors investigate the clustering of nuclear pores in the C. elegans germline and embryo, and explore how this clustering relates to P granule association with the pores. They demonstrate that FG-Nups are essential for promoting pore clustering and that P granule proteins further reinforce this clustering. Additionally, nascent transcripts appear to enhance the interaction between nuclear pores and P granules. Interestingly, mutants that disrupt pore clustering remain fertile and exhibit only minimal gene misregulation, with histones being one of the few affected gene families.
I have a few relatively minor concerns that should be addressable (see below), but overall, this is an engaging manuscript that is likely to be well received by the field.

Comments for the author

Line 152 - "As previously reported…" - need reference for this sentence.

Line 184 - it is mentioned that GLH-1 is one of three related paralogs that contain FG repeats. Are the paralogous proteins also required for P granule - nuclear pore interactions? The glh-1(FGGdel) mutants seems to be more mild in pore clustering phenotypes than some of the other mutants assessed. Could that be due to partial redundancy between GLH-1 and its paralogs?

© 2025. Published by The Company of Biologists under the terms of the Creative Commons Attribution License (https://creativecommons.org/licenses/by/4.0/).

Fig 3B - the "the degree of clustering" metric used in this and other figures is a bit confusing. Just by eye, the nuclear pore clusters in the deltaFGG mutant are much larger and more wild-type looking, compared to the mip mutants. It is unclear what this is measuring if it is not size/distribution of pores. Relatedly, lines 194-198 discuss the relationship of degree of clustering to P granule coverage - again, what can be seen by eye actually correlates quite well with the P granule coverage metric, so I'm not convinced but the data shown that there is a disproportional loss of P granule coverage relative to pore clustering.

Fig 4 - text and images are quite pixelated in some figures (i.e. 4, S4, S6). I'm assuming this is just the version for the reviewers, but I recommend ensuring the quality is high prior to publication

Fig S4E - images are too small/dim to conclude that P granules not affected by nup214(del) in mitotic zone.

Fig 5 - Some quantification of perinuclear vs cytoplasmic localization would help here. For all the germ granule proteins assessed (GLH-1, PRG-1, WAGO-4, MUT-16, CSR-1), there is still some perinuclear localization in the nup214 and nup214 mip-1 deletion mutants. At the same time, there is an increase in cytoplasmic/rachis granules. It is not clear why CSR-1/D granules are not thought to be affected by loss of pore clustering if the localization is altered.

Fig S5E - the result that P granules and Z granules merge in the nup214(del) animals is quite interesting. Some insets (possibly with a different choice of colors) might show this data more convincingly.

Lines 310-311 - "…observed similar levels of sRNAs in each Argonaute class in nup214Δ mip-1Δ animals compared to wild-type (Fig. S6C)." I'm assuming that this is comparing the small RNAs mapping to the set of target genes for each Argonaute in wild-type vs nup-214(del) mip-1(del), but not clear from the text. Rephrase.

Lines 340-343 - sentence mentions absence of granule accumulation in gp210(del), but that data does not appear in the cited figure 7A, and 7C appears to have mRNA expression data which does not directly address granule accumulation.

Lines 473-506 - This section is concluding that Mutator foci can access the nuclear periphery independent of P granules - I don't think this conclusion can be made without colocalzation of MUT-16 and P granules in the mip-1 deletion. It is a bit surprising that the number of perinuclear Mutator foci is not more severely affected in the mip-1 mutant. It would be very important to see whether all the perinuclear Mutator foci in mip-1(del) are also associated with other germ granule proteins. This same section and model figure indicate that D granules stay associated with nuclear periphery in the absence of P granules - but CSR-1 is at large granules at the nuclear periphery and in rachis in nu214(del) and mip-1 nup214(double del). So it would be more accurate to say that D granules, like P bodies, stay associated with both the nuclear periphery and the P granule when they become detached.

Lines 548-551 - I think one could argue that the csr-1 RNAi in the nup214 mip-1 (double deletion) shows an enhancement of the histone mRNA accumulation phenotype relative to the double deletion mutant alone. That could argue that CSR-1 is involved. It is also curious that csr-1, ego-1 and drh-3 mutants have enlarged P granules that I believe also detach from the nuclear periphery. It should be addressed in the discussion whether this might be a related phenotype to what is seen in this manuscript.

First revision

<u>Author response to reviewers' comments</u>

Comments from the Reviewers:

© 2025. Published by The Company of Biologists under the terms of the Creative Commons Attribution License (https://creativecommons.org/licenses/by/4.0/).

Reviewer 1: This study by Thomas, Bodas, and Seydoux, titled "FG repeats drive co-clustering of nuclear pores and P granules in the C. elegans germline," substantiates several hypothesized roles of FG-repeat proteins in the context of nuclear pores and germ granules. It also presents novel findings addressing whether P granule functions depend on their localization at the nuclear periphery. The conclusions are supported by robust and well-controlled evidence. The number of precision-edited C. elegans lines generated is impressive, and the imaging quality is exceptional. The study's findings will significantly impact and interest researchers studying germ granules, the in vivo organization of biomolecular condensates, and small RNA regulation. The following critiques suggest minor revisions to help clarify and further strengthen the study's conclusions.

Line 111 - Soften language - saying the activity is fully dispensable is a high bar.

We have softened the language from “dispensable” to “may not be required”.

Line 234 - The authors may want to speculate on why these clustering observations are so different between the mitotic and pachytene cells.

We speculate in the Discussion (starting in line 436) that nascent transcripts enhance nuclear pore clustering. Since transcription throughout the germline is highest in pachytene, this may provide an explanation for the differences between the mitotic zone and the pachytene region.

Line 241 - The term "partially independent" is hard to interpret and implies that the results could also be "partially dependent."

We have removed the term “partially dependent”.

Line 248 - The initial reference to D granules might pose a challenge since the Huang paper is still on BioRxiv, and many readers may not recognize that D granules refer to DDX-19 granules located at the interface between NPCs and P granules. It may be helpful to provide a broader explanation here. This issue arises again in Figure 5A, where CSR-1 is labeled as a D-granule marker. In the Huang paper, multiple genes are associated with P granules and additional granules, with CSR-1 being just one example. However, at this stage, it seems premature to classify CSR-1 as a D-granule component. The observed retention of some CSR-1 at the nuclear envelope could be explained by its association with P and D granules, but labeling it definitively as a D-granule component does not seem entirely accurate.

The Huang paper is now published in Developmental Cell, so we have updated the references to indicate this. In describing Figure 5A (starting in line 252), we now include a broader explanation for our use of CSR-1 as a P and D granule marker: “The Argonaute CSR-1 has been recently described as a marker for both P and D granules, as it partially overlaps with the D granule marker DDX19 and remains perinuclear in a *mip-1Δ* mutant (Huang et al. 2024). We found that CSR-1 localized to cytoplasmic granules (likely P granules) in *nup214Δ* and *nup214Δ mip-1Δ* mutants and also maintained robust localization at the nuclear periphery (Fig. 5A, 5B, and S5C).” We additionally found that DDX19 behaved similarly to CSR-1 in the *nup214Δ* mutant.

Line 263 - Indicate this as eIF4EIFE-3 as IFE-1 and IFE-3 don't completely overlap.

We have indicated this P-body marker as $eIF4E^{IFE\text{-}3}$ in line 258 and throughout the text as suggested.

Line 272 and S5E - This may require a center-of-mass analysis instead of colocalization, which could be performed using the same images, as P and Z granules show partial overlap in wild-type

© 2025. Published by The Company of Biologists under the terms of the Creative Commons Attribution License (https://creativecommons.org/licenses/by/4.0/).

(WT) conditions. This overlap might be due to exposure or differences in the reporters. Additionally, the sizes of these granules are likely quite different when detached. A center-of-mass analysis would offer a clearer understanding of the extent to which P and Z granules overlap when detached. I would also be interested in hearing the authors speculate on the significance of this observation.

We have now included a center-of-mass analysis for Figure S5G (originally Figure S5E), which indeed shows a greater overlap between PGL-1 and ZNFX-1 in the *nup214Δ* mutant. We have also kept the original overlap analysis as we believe that this measurement reflects the high overlap between PGL-1 and ZNFX-1 that has been reported in wild-type animals (Ishidate et al. 2018, Toraason et al. 2020).

We speculate in the Discussion on a possible reason for why the granules overlap more in the *nup214Δ* mutant (starting in line 473), but prefer not to speculate on the significance of this observation, since we do not have any data that address this issue.

Line 295, 6, and S6A - Rochester et al. (2022) identified the same three classes of genes—sperm, stress (HSP-16), and replication-dependent histones—as being enriched in their analysis of GLH-1-dependent differential translation (polysome vs. total mRNA; see their Fig. 1B and Supplemental Table). While it is true that replication-dependent histones can be overlooked with polyA selection, sufficient sequencing depth can still detect enough of them for differential expression analysis.

We have added this finding and reference in the text, starting in line 296.

Fig S2B - Specify that these are pachytene

We have added a label to the figure and denoted in the legend that these are pachytene germlines.

Figure 4C - The nup214 deletion micrographs appear to show additional nuclear pores that are not marked with white arrows. The authors should clarify the criteria used to identify and label nuclear pores in these images.

We have added additional arrows to Figure 4C in areas where nuclear pores appear. We have also clarified our criteria for identifying nuclear pores in the Materials and methods.

Figure 6 heading. Consider removing glh-1FGG from the heading as it doesn't seem expression profiling was done on this mutant to show elevated levels of histone transcripts

We have removed *glh-1ΔFGG* from the heading for Figure 6 as suggested.

Fig 7B - Quantification is of rachis images, but images are not shown. Because 7A shows mitotic images, it may be incorrectly assumed that the quant in B is for these mitotic images.

We have now noted in the Figure 7B legend that this quantification was performed using pachytene germline images shown in Figure S7D.

Fig 7E - change axis and legend - should not be viability %, but lethality ratio. We have changed the Y axis for Figure 7E to lethality ratio as suggested.

Reviewer 2: SUMMARY OF THE ADVANCE MADE IN THIS PAPER AND ITS POTENTIAL SIGNIFICANCE TO THE FIELD

© 2025. Published by The Company of Biologists under the terms of the Creative Commons Attribution License (https://creativecommons.org/licenses/by/4.0/).

In this manuscript by Thomas et al., the authors investigate the clustering of nuclear pores in the C. elegans germline and embryo, and explore how this clustering relates to P granule association with the pores. They demonstrate that FG-Nups are essential for promoting pore clustering and that P granule proteins further reinforce this clustering. Additionally, nascent transcripts appear to enhance the interaction between nuclear pores and P granules. Interestingly, mutants that disrupt pore clustering remain fertile and exhibit only minimal gene misregulation, with histones being one of the few affected gene families.
I have a few relatively minor concerns that should be addressable (see below), but overall, this is an engaging manuscript that is likely to be well received by the field.

SUGGESTIONS TO AUTHORS

Line 152 - "As previously reported..." - need reference for this sentence.

We have now cited Sheth et al 2010.

Line 184 - it is mentioned that GLH-1 is one of three related paralogs that contain FG repeats. Are the paralogous proteins also required for P granule - nuclear pore interactions? The glh-1(FGGdel) mutants seems to be more mild in pore clustering phenotypes than some of the other mutants assessed. Could that be due to partial redundancy between GLH-1 and its paralogs?

We have added the point about likely redundancy between the GLH paralogs in the Discussion (line 426): “Studies have shown that GLH-1 functions redundantly with GLH-2 and GLH-4, which also have FG repeats (Spike et al. 2008; Marnik et al. 2019; Chen et al. 2020); redundancy may explain why the *glh-1ΔFGG* mutation has a comparatively mild effect on P granule nuclear association.”

Fig 3B - the "the degree of clustering" metric used in this and other figures is a bit confusing. Just by eye, the nuclear pore clusters in the deltaFGG mutant are much larger and more wild-type looking, compared to the mip mutants. It is unclear what this is measuring if it is not size/distribution of pores. Relatedly, lines 194-198 discuss the relationship of degree of clustering to P granule coverage - again, what can be seen by eye actually correlates quite well with the P granule coverage metric, so I'm not convinced but the data shown that there is a disproportional loss of P granule coverage relative to pore clustering.

We previously used the “degree of clustering” metric to measure the standard deviation of grey values per nucleus. The standard deviation will be higher from nuclei with larger clusters, corresponding large gaps between clusters. However, this metric may be less sensitive at measuring smaller differences in cluster size (as in Figure 3B), so we have switched to using a measurement of cluster size for this figure. With this new measurement, we see a trend in which clusters are largest in wild-type, followed by *glh-1ΔFGG*, then *mip-1Δ*, and finally the *mip-1Δ mip-2Δ* double mutant. This mirrors the NDC1 area overlapped by PGL-1 also measured in this figure.

We have updated the description of this figure in line 189 as follows: “Nuclear pore clustering was also reduced in the mutants, in a manner proportional to the loss of coverage by P granules (Fig. 3B).”

Fig 4 - text and images are quite pixelated in some figures (i.e. 4, S4, S6). I'm assuming this is just the version for the reviewers, but I recommend ensuring the quality is high prior to publication

We apologize, this may be due to the compression of large files. We will submit high-resolution figure files for the re- submission.

© 2025. Published by The Company of Biologists under the terms of the Creative Commons Attribution License (https://creativecommons.org/licenses/by/4.0/).

Fig S4E - images are too small/dim to conclude that P granules not affected by nup214(del) in mitotic zone.

This may also be due to the pixelated images in the figures and will hopefully be more apparent with the submission of high-resolution figure files. Nonetheless, we have softened the language in line 216 to say: “In *nup214Δ* mutants, P granule association with nuclei was reduced in the pachytene region but appeared wild-type in the mitotic zone”.

Fig 5 - Some quantification of perinuclear vs cytoplasmic localization would help here. For all the germ granule proteins assessed (GLH-1, PRG-1, WAGO-4, MUT-16, CSR-1), there is still some perinuclear localization in the nup214 and nup214 mip-1 deletion mutants. At the same time, there is an increase in cytoplasmic/rachis granules. It is not clear why CSR-1/D granules are not thought to be affected by loss of pore clustering if the localization is altered.

We have now included quantification of the percent of granules in the cytoplasm/rachis for GLH-1 (P granules), WAGO-4 (Z granules), MUT-16 (Mutator foci), and CSR-1 (P and D granules) in Figure S5C. This quantification shows that GLH-1, WAGO-4, and MUT-16 are more severely affected in the *nup214Δ mip-1Δ* double mutant, with ~40-70% of granules accumulating in the rachis. In contrast, only ~20% of CSR-1 granules accumulate in the rachis, likely corresponding to the P granule-localized pool of CSR-1.

We have discussed new quantification starting in line 255: “We found that CSR-1 localized to cytoplasmic granules (likely P granules) in *nup214Δ* and *nup214Δ mip-1Δ* mutants and also maintained robust localization at the nuclear periphery (Fig. 5A, 5B, and S5C).”

Fig S5E - the result that P granules and Z granules merge in the nup214(del) animals is quite interesting. Some insets (possibly with a different choice of colors) might show this data more convincingly.

We have provided enlarged insets in the new Figure S5G (originally Figure S5E) showing just PGL-1 (P granules) and ZNFX-1 (Z granules) for clarity.

Lines 310-311 - "…observed similar levels of sRNAs in each Argonaute class in nup214Δ mip-1Δ animals compared to wild-type (Fig. S6C)." I'm assuming that this is comparing the small RNAs mapping to the set of target genes for each Argonaute in wild-type vs nup-214(del) mip-1(del), but not clear from the text. Rephrase.

We have rephrased this sentence as suggested starting in line 307: “We sequenced sRNAs and observed similar levels of sRNAs mapping to target genes for each Argonaute class in *nup214Δ mip-1Δ* animals compared to wild-type (Fig. S6C).”

Lines 340-343 - sentence mentions absence of granule accumulation in gp210(del), but that data does not appear in the cited figure 7A, and 7C appears to have mRNA expression data which does not directly address granule accumulation.

We have removed the *gp210Δ* mutant mRNA expression data from Figure 7C.

Lines 473-506 - This section is concluding that Mutator foci can access the nuclear periphery independent of P granules - I don't think this conclusion can be made without colocalzation of MUT-16 and P granules in the mip-1 deletion. It is a bit surprising that the number of perinuclear Mutator foci is not more severely affected in the mip-1 mutant. It would be very important to see whether all the perinuclear Mutator foci in mip-1(del) are also associated with other germ granule proteins. This same section and model figure indicate that D granules stay associated with nuclear periphery in the absence of P granules - but CSR-1 is at large granules at the nuclear periphery and in rachis in nu214(del) and mip-1 nup214(double del). So it would be more accurate to say that D

© 2025. Published by The Company of Biologists under the terms of the Creative Commons Attribution License (https://creativecommons.org/licenses/by/4.0/).

granules, like P bodies, stay associated with both the nuclear periphery and the P granule when they become detached.

We have rewritten this section as suggested and removed the suggestion that Mutator foci can access the nuclear periphery independent of P granules.

Lines 548-551 - I think one could argue that the csr-1 RNAi in the nup214 mip-1 (double deletion) shows an enhancement of the histone mRNA accumulation phenotype relative to the double deletion mutant alone. That could argue that CSR-1 is involved. It is also curious that csr-1, ego-1 and drh-3 mutants have enlarged P granules that I believe also detach from the nuclear periphery. It should be addressed in the discussion whether this might be a related phenotype to what is seen in this manuscript.

We now cite the enlarged P granule phenotype of *csr-1* and other mutants in the Discussion. We do not know whether the apparent enhancement of histone mRNA accumulation in P granules by *csr-1* RNAi is a visual artefact due to *csr-1*'s effect on P granules (which become fused and larger in *csr-1* mutants), or a direct effect on histone mRNAs, or a combination. We explain this in the legend to Figure S7F.

Second decision letter

MS ID#: dev.204585R1

MS Title: FG repeats drive co-clustering of nuclear pores and P granules in the C. elegans germline

Authors: Laura L. Thomas; Devavrat Bodas; Geraldine Seydoux
Article Type: Research Article

I am happy to tell you that your manuscript has been accepted for publication in Development, pending our standard publication integrity checks.

Reviewer 1

Advance summary and potential significance to field

-

Reviewer 2

I am satisfied that my recommendations have been appropriately addressed.

© 2025. Published by The Company of Biologists under the terms of the Creative Commons Attribution License (https://creativecommons.org/licenses/by/4.0/).
